# Prediction of deleterious mutations in coding regions of mammals with transfer learning

**DOI:** 10.1111/eva.12607

**Published:** 2018-05-09

**Authors:** Elena Plekhanova, Sergey V. Nuzhdin, Lev V. Utkin, Maria G. Samsonova

**Affiliations:** ^1^ Peter the Great St. Petersburg Polytechnic University St. Petersburg Russia; ^2^ Program Molecular and Computation Biology Dornsife College of Letters, Arts, and Sciences University of Southern California Los Angeles CA USA

**Keywords:** classification, deleterious mutations, transfer learning

## Abstract

The genomes of mammals contain thousands of deleterious mutations. It is important to be able to recognize them with high precision. In conservation biology, the small size of fragmented populations results in accumulation of damaging variants. Preserving animals with less damaged genomes could optimize conservation efforts. In breeding of farm animals, trade‐offs between farm performance versus general fitness might be better avoided if deleterious mutations are well classified. In humans, the problem of such a precise classification has been successfully solved, in large part due to large databases of disease‐causing mutations. However, this kind of information is very limited for other mammals. Here, we propose to better use information available on human mutations to enable classification of damaging mutations in other mammalian species. Specifically, we apply transfer learning—machine learning methods—improving small dataset for solving a focal problem (recognizing damaging mutations in our companion and farm animals) due to the use of much large datasets available for solving a related problem (recognizing damaging mutations in humans). We validate our tools using mouse and dog annotated datasets and obtain significantly better results in companion to the SIFT classifier. Then, we apply them to predict deleterious mutations in cattle genomewide dataset.

## 1 INTRODUCTION

The amount of data gathered in biological experiments grows at an alarming rate, especially in the field of genomics. However, the field of knowledge as a whole remains fractured, which is an acute problem. Consequently, one of the questions of comparative genomics is: When we have a few well‐studied model organisms, how can we transfer this knowledge to phylogenetically related organisms? Indeed, cross‐referencing the information from connected fields of science and art is a common practice. For a real‐life example, consider a person who plays the violin and desires to learn the piano. It would be easier for them to learn to play piano than for a person without any experience with a musical instrument. Similar transfers of knowledge are sometimes used to solve classification and regression problems. This process is formalized in machine learning techniques called “transfer learning.”

Here, we consider the application of transfer learning to the problem of classification of mutations into categories “deleterious” versus “neutral.” This problem is interesting for both theoretical and practical considerations. Not only can we use this knowledge to explore functional genome variation in multiple species, but we can also study their evolution, ultimately with an aim to understand why a mutant allele classified as deleterious in one species may be in fact a normal allele in another species (Charlesworth, 2012; Hartfield, Glémin, Yu, & Purugganan, 2014; Kaiser & Charlesworth, 2009). Further, enhanced understanding of deleterious genomic content may enhance selection and breeding in crop species and may also improve veterinary medicine capabilities in species such as cattle, pigs, and dogs. For example, Raszek, Guan, and Plastow (2016) describe the application of comparative genomic analysis for treatment of infectious disease and understanding of developmental abnormality, which is a major economic burden in cattle production worldwide. Similarly, (Knol, Nielsen, and Knap (2016) consider the application of functional, knowledge‐enabled genomic selection in commercial pig breeding.

Here, we utilize the transfer of knowledge from the classification of human mutations to similar categorizations in other mammalian species. This direction of knowledge transfer was chosen because of the abundance of both information about the properties of human mutations (Capriotti, Calabrese, & Casadio, 2006; Kondrashov, 2012) and inferences from association studies (MacArthur et al., 2017), and relatively small amount of information of mutations in other species. Firstly, we train a classifier on a labeled human data. Further, we use a relatively small amount of information from other species to reduce any biases due to human‐centric training when applied to other mammals, particularly to mouse, dog, and cattle.

Formally, we consider deleterious mutations as a genetic alteration that increases an individual's susceptibility or predisposition to a certain disease or disorder. Nearly all deleterious mutations in nonhuman mammals are found in the coding part of the genome and are typically missense mutations, that is, those which cause amino acid changes in the corresponding protein. However, many missense mutations do not cause a disease (Huber, Kim, Marsden, & Lohmueller, 2017; Kim, Huber, & Lohmueller, 2017). Here, we classify mutations into one of two classes: “deleterious” or “neutral.” To accomplish this task, there are a number of characteristics that need to be known about the mutation, such as whether it is a transition or a transversion (Stoltzfus et al., 2016), and the frequency of a particular mutation in the population (a detailed description of these characteristics is available in the Methods section). Thus, for each mutation, there are a number of features for classification.

There are two main ways to perform classification. Firstly, the values of the features can be compared with some threshold. For example, this concept was implemented in SIFT (Sim et al., 2012) and PolyPhen‐1 (Ramensky, Bork, & Sunyaev, 2002). SIFT, in particular, uses the conservation score of the sequence as the threshold. Secondly, classification can be performed using machine learning techniques. In this case, the set of labeled mutations is used as a training set for a classifier, and then, the trained classifier is applied to make predictions on an unlabeled set. The advantage of machine learning methods is that parameters do not have to be manually set, but can instead be identified automatically based on the labeled set. This concept was realized in PolyPhen‐2 (Adzhubei et al., 2010), where the classification consists of two steps. The first step is feature preparation: The software collects necessary information about a mutation using sequence characteristics, multiple alignment scores, and information about the 3D structure of the resulting protein. The second step is classification by applying a naïve Bayes approach. In this work, we use PolyPhen‐2′s preparation step; however, the second step, the classification of data in a species of interest, was optimized.

A substantial amount of research has been devoted to classification of human mutations (Pabinger et al., 2014). However, only two well‐known programs are useful for classification of mutations in nonhuman mammals: SIFT (Sim et al., 2012) and MAPP (Stone & Sidow, 2005). They both are based on evolutionary conservation of polymorphism inferred through multiple alignment scores (MAPP also uses a phylogenetic tree) to make a prediction. While their predictions are reasonably accurate, both SIFT and MAPP might potentially be improved by taking advantage of additional classification features, such as the 3D structure of the resulting protein, or properties of already classified mutations. Further, their performance might be enhanced with incorporation of machine learning techniques, for instance, choosing the thresholds automatically, as PolyPhen‐2 does for classification of human mutations.

There is, in principle, an option in PolyPhen‐2 for classification of mutations in nonhuman species. However, it is an experimental feature and it makes predictions using a classifier trained on human data. In our work, we aim to modify this procedure and use transfer learning techniques in order to improve the accuracy of the classification via retraining on species‐specific, although limited, datasets.

An assumption of traditional machine learning is that the training data and testing data are taken from the same domain, such that the input feature space and data distribution characteristics are the same. However, in some real‐world machine learning scenarios, this assumption is unfeasible because training data are expensive or difficult to collect (Weiss, Khoshgoftaar, & Wang, 2016). Therefore, there is a need to create high‐performance learners trained with more easily obtained data from different domains. The methodology addressing this need is referred to as transfer learning.

The transfer learning problem can be formally defined as follows. Let domain *D *= {*X*,* P*(*X*)} be characterized by two parts: a feature space *X* and a marginal probability distribution *P*(*X*). For a given domain *D*, a task *T = {Y, f*(·)} is defined by two parts: a label space *Y*, and a predictive function *f*(·), which is learned from labeled features. Now, *D*_*s*_ *, T*_*s,*_ *f*_*s*_(·) are referred to as the source dataset, source task, and source predictive function, respectively, and *D*_*t*_ *, T*_*t,*_ *f*_*t*_(·) are referred to as the target dataset, target task, and target predictive function, respectively. Transfer learning is the set of machine learning methods that aim to improve the target predictive function *f*_*t*_(·) using related information from the source dataset *D*_*s*_ and source task *T*_*s*_, where *D*_*s*_ ≠ *D*_*t*_ or *T*_*s*_ ≠ *T*_*t*_.

Transfer learning methods are actively used for a large variety of real‐world tasks from atmospheric dust aerosol particle classification to face motion recognition (Kan, Wu, Shan, & Chen, 2014; Ma, Gong, & Mao, 2015; Perlich, Dalessandro, Stitelman, Raeder, & Provost, 2013). There are several applications of transfer learning methods in bioinformatics, for example, for recognition of splicing sites (Schweikert, Schweikert, & Widmer, 2008; Widmer, Leiva, Altun, & Rätsch, 2010), gene expression analysis (Chen & Huang, 2010; Q. Xu, Xue, & Yang, 2011), and image recognition (Bi, Xiong, Yu, Dundar, & Rao, 2008). However, despite a growing amount of SNP data, only a few studies have applied transfer learning to SNP‐related analyses (Chen & Huang, 2010; Puniyani, Kim, & Xing, 2010; Xu et al., 2011).

Different techniques of transfer learning are best used depending on the research needs (Weiss et al., 2016). We want to use both the labeled source dataset of human mutations and the small labeled target datasets of mouse, dog, or cattle mutations to better predict unlabeled mutations in these nonhuman species. So, we assume that source data are labeled and target data are unlabeled or partly labeled. Under these circumstances, one should use transductive and inductive transfer learning techniques.

Transductive transfer learning is an example of instance transfer based on reweighting samples of source data according to their distances to target data. (Chattopadhyay et al., (2012) proposed to use this approach while working with multiple labeled source domains. The main idea is to use a combination of source domain classifiers, with weights assigned as a function of the closeness in conditional distribution between each source and target domain, to label the unlabeled target data. Other authors have used different sample weights metrics to account for instances from one source domain (Xu et al., 2017).

The inductive transfer learning solution belongs to feature‐based transfer learning approaches. It transforms the feature spaces of source and target data to make them more similar and, thus, to decrease classification error. The transformation of source data to a new feature space can be implemented with a denoising autoencoder (Glorot, Bordes, & Bengio, 2011), or convolutional neural networks (Oquab, Bottou, Laptev, & Sivic, 2014).

Here, we firstly demonstrate the applicability of transductive and inductive transfer learning techniques for the prediction of deleterious mutations in dog and mouse datasets and then apply transductive transfer learning to predict deleterious mutations in cattle.

## 2 MATERIALS AND METHODS

### 2.1 Datasets

To apply machine learning technique to the problem of mutation classification, we needed to create labeled (deleterious versus neutral) datasets of mutations in order to train classifiers and assess their performance. Sets of deleterious mutations are usually compiled from databases with a focus on disease‐causing mutations. Sets of neutral mutations are composed of nonsynonymous single‐nucleotide mutations that satisfy one of two criteria: They have been fixed during divergence between the species of interest and its closely related species, or their MAF in population is at least 5% (Ng & Henikoff, 2001). More detailed information on the datasets used as well as on how they are created is presented below.

#### 2.1.1 Human datasets

We used the two standard human datasets, HumDiv and HumVar, that are applied by PolyPhen‐2, to train our classifier. The HumDiv dataset (Adzhubei et al., 2010) contains 5564 mutations from the UniProtKB database that are known to cause Mendelian diseases and a set of 7,539 DNA variants between human proteins and their closely related mammalian homologs that are assumed to be nondamaging. The HumVar dataset (Capriotti et al., 2006) consists of 22,196 human disease‐causing mutations from UniProtKB and 21,151 neutral mutations that are common human nsSNPs (MAF>1%) without annotated involvement in disease. Note that the HumDiv dataset contains annotated mutations directly associated with human diseases, while the HumVar dataset is more noisy as its neutral mutation subset includes many mildly deleterious alleles.

#### 2.1.2 Mouse dataset

We used both the MGI database (Eppig et al., 2017) and the Disease Ontology Database (Kibbe et al., 2015) to retrieve a set of 189 amino acid substitutions associated with different mouse diseases.

A set of 188 neutral mutations was compiled from nonsynonymous SNPs present in 28 strains of mouse. We selected positions that were annotated in more than eight *Mus* strains and that were different in no less than two strains. Thus, in total the dataset consists of 377 mouse mutations, of which 189 are deleterious and 188 are neutral (see Table S1).

#### 2.1.3 Dog and cattle62 datasets

The dog and cattle (called cattle62) datasets were generated similarly. To retrieve deleterious mutations, we used the OMIA database (Online Mendelian Inheritance in Animals) (Lenffer et al., 2006) containing information on deleterious mutations in animals. We considered only Mendelian diseases with known key missense mutation.

The sets of neutral mutations were generated from nonsynonymous single‐nucleotide mutations in homologous proteins using the UniProtKB database (Consortium, 2017). From this database, only the entries that were reviewed by database curators were selected. Then, for each entry, the following procedure was applied:

1. From a set of sequences similar to a query protein and retrieved with BLASTp (Altschul et al., 1997), only mammalian proteins with more than 95% identity to the input sequence were selected.
2. Clustal Omega (Sievers et al., 2014) was used to construct multiple alignments for sequences selected in the previous step.
3. The only amino acid substitutions considered were those that were isolated (i.e., not present in a continuous block of substituted residues) and independent (i.e., there were no other substitutions in the same sequences of alignment). This helps us to avoid the phenomenon of correlated mutational behavior between columns of a multiple sequence alignment (Kowarsch, Fuchs, Frishman, & Pagel, 2010). In addition, we chose only substitutions present in no less than two aligned sequences.

The generated dog dataset consists of 207 mutations, of which 103 are deleterious and 104 are neutral. For cattle62, we retrieved a set of 62 mutations, of which 30 were deleterious and 32 neutral (see Table S1).

#### 2.1.4 CattleGW dataset

The cattle genomewide (CattleGW) dataset was created using the Ensembl database. We found approximately 100,000,000 different genetic variants in cattle, almost 2,000,000 of which were missense mutations in 21,599 transcripts corresponding to 21,370 proteins in the UniProtKB database. For each mutation, we collected information about its position within the protein, type of amino acid substitution, and SIFT prediction score. We used PolyPhen‐2 to prepare features for further classification of 1,892,964 mutations in 18,716 proteins (see Table S2).

### 2.2 Feature description

To make predictions about the effects of nonsynonymous SNPs on protein function, we need to characterize these substitutions with informative predictive features. We used 14 predictive features (Table 1), of which 11 are standard predictive features used by the PolyPhen‐2 algorithm (Adzhubei et al., 2010). Three additional features were added to improve final classification: Grantham score, which predicts evolutionary distance between two amino acids (Grantham, 1974); BLOSUM62 substitution score (Henikoff & Henikoff, 1992); and PDB_id, which is an indicator of the availability of 3D structure information.

**Table 1:** Description of features used in classification

Feature name	Description^a^
GScore	Grantham score
BScore	Amino acid score in BLOSUM62 matrix
Score1	PSIC score (Sunyaev et al., 1999) for wild‐type amino acid residue (before substitution)
dScore	Difference of PSIC (Sunyaev et al., 1999) scores for two amino acid residue variants (before and after substitution)
Nobs	Number of residues observed at the substitution position in multiple alignment (without gaps)
NormASA	Normalized accessible protein surface area
dVol	Change in residue side chain volume
dProp	Change in solvent accessible surface propensity resulting from the substitution
B.fact	Normalized B‐factor (temperature factor; Chasman & Adams, 2001) for the residue
IdPmax	Maximum congruency of the mutant amino acid residue to all sequences in multiple alignment
IdQmin	Query sequence identity with the closest homolog deviating from the wild‐type amino acid residue
PDB_id	Availability of PDB (Berman et al., 2000) protein structure identifier
Transv	Substitution type (transversion or transition)
PfamHit	Availability of Pfam (Finn et al., 2016) protein structure identifier

a A more detailed description is available at http://genetics.bwh.harvard.edu/pph2/dokuwiki/appendix_a.

In all of the datasets, all predictive feature values were centered and normalized. Missing values were imputed with median values.

### 2.3 Classifiers

We used the following set of different classifiers, which includes probabilistic ones: Naïve Bayes (NB) with Gaussian kernel, Boosted Naïve Bayes classifier (Freund & Schapire, 1999), Logistic Regression, Support Vector Machines (SVM) (Cortes & Vapnik, 1995) with three different kernels (linear, Gaussian and polynomial), Random Forest (Breiman, 2001), Neural Network (Jantzen, 1998), and a relatively new approach called Deep Forest (Zhou & Feng, 2017). We used two performance measures: AUC, which is the area under a ROC curve, and accuracy (acc), which is the proportion of correctly classified samples. The classes were balanced so that the accuracy metrics worked correctly.

We trained and tuned the classifiers on the HumDiv and HumVar datasets. To avoid overfitting, fivefold cross‐validation was performed. Specifically, a dataset was split into five approximately equal parts (or folds), of which four parts were used for training and the fifth part for validation. This procedure was repeated five times with different parts used for validation each time. The performance measure is an average of the values computed at each iteration. While the dataset is usually split into folds randomly, we created folds such that all mutations in the same protein fell into the same fold. This was done to avoid overfitting in the situation where we train and test a classifier on the same protein.

The grid search method guided by the accuracy metric was used to tune classifiers (see best parameters in Table S3).

To construct classifiers, we used the GaussianNB, LogisticRegression, LinearSVC, SVC, RandomForestClassifier, MLPClassifier, and AdaBoostClassifier functions of the sklearn package (Pedregosa et al., 2011) in Python 3.1. To calculate acc, AUC scores, and ROC curves, we used the roc_curve, auc, and accuracy_score functions of the sklearn metrics module. We used the sklearn model_selection module to train and test classifier performance, search for the best parameters, and perform cross‐validation.

### 2.4 Transfer learning techniques

As source and target dataset distributions differ (see Figure S1), direct predictions of deleterious mutations in target datasets using classifiers trained on source data could be biased. Therefore, we applied transfer learning techniques to classify mutations in target datasets. To transfer knowledge from classification of human mutations to similar tasks for other mammals, we applied two different approaches: transductive transfer learning and inductive transfer learning.

#### 2.4.1 Transductive transfer learning

Transductive transfer learning assumes that the source data are labeled but target data are not. So, we did not use labels for the target data until final validation of the predictions. We tested and tuned classifiers on human datasets, and the parameters that provided the best classifier performance were further used in transfer learning tasks (see Table S3 for details). Each sample from the source data

$$x_{i}^{s}$$

was multiplied by weight *w*_i_ that inversely depends on the distance from this sample to the mean of the cluster of the target data a:

$$w_{i}=\text{exp}\;\left(-{\left(x_{i}^{s}-a^{T}\right)}^{2}\right)$$

Therefore, samples from source data were taken into account only if they are close to the target data. This method prevents the negative transfer effect, that is, the situation when samples from source data not connected with the target task are used thereby degrading the classifier performance. The classifiers are learned based on reweighted source data and tested on target data. To assess the performance of transfer learning strategy, we compared its results with the results of direct classification without weights.

#### 2.4.2 Inductive transfer learning

Inductive transfer learning assumes that source data and target data are labeled. Accordingly, we used labels for target data to construct a classifier and to test its performance. To provide independent test samples, we used cross‐validation (see the “Classifiers” subsection for a description of how the folds were made).

The feature representation transfer is an instance of the transductive transfer learning strategy that aims to transform the feature spaces of the source and target data to make them more similar and, thus, to decrease classification error. The new feature representation space for the target data was found by applying a neural network trained on the source data. The algorithm works as follows. First, we began with a neural network trained on source data, found parameters and weights, and fixed them. Next, the neural network was pruned at the last layer, which is just before the classification layer, and the target data were passed through the obtained network. Finally, the transformed target data in the new feature space were split into two parts, one for training and one for testing. Each classifier was trained on the training set and tested on the test set.

We used a three‐layered network with 200, 200, and 15 nodes in the first, second, and third layers, respectively. The rectifier activation function and hyperbolic tangent function were used for the first two layers and the last layer, respectively. The training and testing procedures were repeated 30 times to obtain statistically significant results. The Wilcoxon–Mann–Whitney test was applied to compare classification results of the target data in the initial feature space to those of the new space.

We validated the inductive transfer learning approach on mouse data using the HumVar dataset as source data. The size of this dataset is sufficient to train a neural network. We used mouse data to validate this learning strategy as dog data are almost linearly separable and therefore can be predicted with a few samples. In such situations, information learned from a source domain may lead to a negative transfer effect.

#### 2.4.3 Implementation characteristics

We used Python 3.1 to write all the scripts and the sklearn package (Pedregosa et al., 2011) to implement machine learning tasks. Sample reweighting was performed with the standard argument “sample_weight” of the fit() attribute of the classifiers. A neural network was implemented using sknn (https://github.com/aigamedev/scikit-neuralnetwork). SIFT BLINK (Sim et al., 2012) was used as a standard in comparisons of the classifier accuracy developed on mouse and dog data. The implementation of transfer learning algorithm is available as supplementary code at https://github.com/PlekhanovaElena/Transfer_learning.

Note that to classify different target datasets, one should retrain the transductive classifier with weights computed from the distance between the source and the target data, which are different each time. However, training a classifier on human data with weights takes a few minutes: 5 min for HumDiv dataset and 15 min for HumVar dataset. In case of using inductive classifier to classify different target dataset, we can train the neural network only once (it takes 2 hr for HumDiv and 8 hr for HumVar) and then pass each target dataset of interest through the obtained network (one pass takes about 15 min).

## 3 RESULTS AND DISCUSSION

To obtain reasonable predictions for the target data, we need to ascertain that a classifier attains significant accuracy on the source data. Therefore, we compared the performance of different classification methods on human data and selected the best classifiers that were further used in transfer learning tasks.

### 3.1 Classification of human data

The human data consist of two datasets, HumDiv and HumVar. The HumDiv dataset (Ramensky et al., 2002) consists of 13,103 mutations that are either neutral with respect to closely related mammalian homologs or cause human Mendelian diseases. HumVar (Capriotti et al., 2006), consisting of 43,347 mutations, treats any disease‐causing mutation as damaging, and it assumes that common human nonsynonymous SNPs (MAF>1%) are neutral, as long as they have not been annotated as disease‐causing.

**Figure 1:**
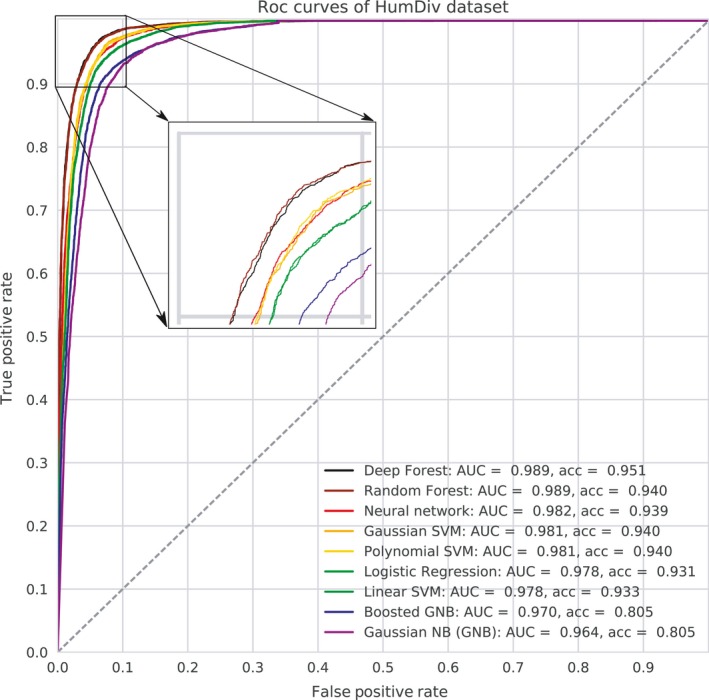
ROC curves for different classifiers, trained on HumDiv data. Values of quality metrics ordered by decreasing AUC values are shown adjacent to the classifier name. The dotted line corresponds to the ROC curve for random guessing. The inset zooms in on the left upper quadrant to better distinguish the ROC curves

We first tested the performance of nine different classifiers: Deep Forest, Random Forest, Neural Network, Gaussian SVM, Polynomial SVM, Linear SVM, Logistic Regression, Gaussian Naïve Bayes, and Boosted Gaussian Naïve Bayes. The quality of each predictor was assessed using two metrics: AUC, which is the area under the ROC curve, and the accuracy rate, which is the fraction of correct predictions. On both datasets, the best results were achieved with Deep Forest, Random Forest, Neural Network, Polynomial SVM, Gaussian SVM, Linear SVM, and Logistic Regression (Figure 1, Figure S2). On the HumDiv dataset, the classification errors varied from 5% to 7% and the AUC values ranged from 98% to 99%. On the HumVar dataset, the classification errors were larger ranging from 16% to 18% and the AUC values varied from 89% to 91%. As a result, we keep these classifiers for further analysis.

Next, we compared the classifiers with the Naïve Bayes classifier used by PolyPhen‐2 (Adzhubei et al., 2010) and with the SIFT classifier (Sim et al., 2012). Most classifiers better predicted deleterious mutations than PolyPhen‐2 given that they resulted in larger values of TPR (true‐positive rate) for a given FPR (false‐positive rate) than PolyPhen‐2 (Table 2). Note that the Deep Forest classifier showed the best results, which were significantly better than PolyPhen‐2 predictions. As for the SIFT classifier, it shows results worse than PolyPhen‐2: 0.863 accuracy on HumDiv and 0.796 on HumVar dataset.

**Table 2:** Comparison of classifier performance on human datasets

Classifier	HumDiv				HumVar			
	FPR			acc	FPR			
	0.05	0.10	0.20		0.05	0.10	0.20	
	TPR				TPR			acc
Deep Forest	**0.950**	**0.986**	0.996	**0.951**	**0.584**	**0.749**	**0.876**	**0.842**
Random Forest	0.947	**0.986**	**0.997**	0.940	0.563	0.733	0.868	0.830
Neural Network	0.916	0.972	0.993	0.939	0.548	0.716	0.857	0.827
Gaussian SVM	0.916	0.975	0.995	0.940	0.551	0.719	0.857	0.829
Polynomial SVM	0.917	0.973	0.995	0.940	0.549	0.716	0.854	0.828
Logistic Regression	0.895	0.961	0.992	0.931	0.484	0.666	0.831	0.814
Linear SVM	0.897	0.961	0.992	0.933	0.483	0.667	0.831	0.815
Boosted Gaussian NB	0.850	0.936	0.977	0.805	0.445	0.650	0.822	0.812
Gaussian NB	0.794	0.928	0.978	0.805	0.341	0.568	0.813	0.812
PolyPhen‐2	0.78	0.89	0.96	0.89	0.53	0.68	0.83	0.81

TPRs (true‐positive rates) corresponding to a given FPR (false‐positive rate) are provided. The values of the accuracy metric (acc) are given for all classifiers ordered by decreasing of AUC metric (see Figure 1 and Figure S2). Cells with AUC or acc values no less than corresponding PolyPhen‐2 values are filled in yellow, while cells with AUC or acc values smaller than corresponding PolyPhen‐2 values are colored light blue. In each column, the maximal value is in bold.

### 3.2 Transfer learning for classification of mouse and dog data

Due to the relatively small amount of information on deleterious mutations, the application of traditional machine learning methods to mutation classification in nonhuman species does not seem possible. A good alternative may be to use transfer learning trained with more easily obtained human data. Here, we tested the applicability of two transfer learning methods, namely transductive and inductive transfer learning, to predict deleterious mutations in dog and mice.

The transfer learning methods improve the target data classification using related information from other source datasets. We aim to transfer knowledge from classification of human mutations to similar tasks in dog and mouse and, consequently, we used HumVar and HumDiv as source datasets.

To construct the target dog and mouse datasets, we used UniProtKB (Consortium, 2017) and either the OMIA (Lenffer et al., 2006) or MGI (Eppig et al., 2017) database (see Methods for a more detailed description). Mutations associated with Mendelian diseases are considered damaging. Putative neutral mutations were sampled either from positions that differ in alignments between human proteins and their closely related mammalian homologs or from common variants segregating within species.

#### 3.2.1 Transductive transfer learning

The transductive transfer learning assumes that target data are unlabeled. We applied instance transfer learning to reweight samples from the source dataset according to their distance to the target data. To assess the classification results, we trained each classifier independently on the source data with and without weights and then tested its performance on the target data. For this analysis, we considered only five classifiers, for which reweighing is possible: Random Forest, Polynomial SVM, Gaussian SVM, Logistic Regression, and Linear SVM.

As evident from Table 3, the application of transductive transfer learning does not impair the performance of any classifier and, furthermore, improves the performance of several of them. The best results were reached with Random Forest and Logistic Regression. Improvement was most noticeable in the following source–target data pairs: (i) HumDiv‐dog data, where classification using Logistic Regression was improved by 23.6% in comparison with the best result without using transfer learning; and (ii) HumVar‐mouse data, where improvement of classification with the same classifier is 19.3% in comparison with the best result without using transfer learning.

**Table 3:** Comparison of the quality of classification of deleterious mutations in dog and mouse datasets for classifiers, trained with weights (+TL) and without weights (—) on source data

Classifier	Dog				Mouse			
	Trained on HumDiv		Trained on HumVar		Trained on HumDiv		Trained on HumVar	
	+TL	—	+TL	—	+TL	—	+TL	—
Random Forest	0.855	0.638	**0.889**	0.884	**0.846**	0.682	0.841	0.682
Polynomial SVM	0.874	0.657	0.715	0.454	0.764	0.764	0.812	0.528
Gaussian SVM	0.686	0.662	0.753	0.618	0.655	0.539	0.833	0.560
Logistic Regression	**0.908**	0.667	0.855	0.701	0.777	0.576	**0.875**	0.565
Linear SVM	0.672	0.672	0.715	0.715	0.597	0.597	0.852	0.568

In each cell, we present the accuracy rate reached by a classifier. The maximal accuracy values achieved are shown in bold.

Even though it is the most often used tool to predict deleterious mutations in nonhuman species, we showed that SIFT is significantly less accurate in predicting deleterious mutations in dog and mouse datasets than transfer learning methods. On the dog dataset, SIFT achieves an accuracy of 85.2%, while Logistic Regression trained on the HumDiv dataset has an accuracy of 90.8%. On the mouse dataset, SIFT has an accuracy of 84.9%, while the Logistic Regression classifier trained on the HumVar dataset has an accuracy of 87.5%.

#### 3.2.2 Inductive transfer learning

Inductive transfer learning assumes that the target data are labeled and aims to improve classification of the target data by transforming the feature spaces of both source and target data to make them more similar. Here, we transform the target feature space using a neural network trained on source data. To estimate classification accuracy, each classifier was independently trained either on one‐third of the transformed or initial target data and then tested on the remaining part of the target data. To test this approach, we used the HumVar and mouse datasets as source and target data, respectively (see Methods for the details).

As it is evident from Table 4, the classifiers can reach, on average, 87.9% accuracy in the initial feature space and 88.4% accuracy in the transformed one. For Random Forest, Neural Network, Gaussian SVM, and Logistic Regression, the classification in transformed feature space leads to more accurate predictions according to the Wilcoxon–Mann–Whitney test (*p *<* *.0025) (Wilcoxon, 1945).

**Table 4:** Comparative analysis of classification quality (accuracy) for classifiers trained without transformation (in initial feature space) and with transformation (in transformed space from the use of a neutral network) on mouse data

Classifier	Without transformation		With transformation		*p*‐value^a^
	acc^a^	Conf. interval	acc	Conf. interval	
Deep Forest	**0.879**	(0.873, 0.884)	0.881	(0.877, 0.887)	.160
Random Forest	0.875	(0.869, 0.882)	**0.884**	(0.879, 0.889)	.0014
Neural Network	0.840	(0.830, 0.850)	0.866	(0.854, 0.873)	.0005
Gaussian SVM	0.866	(0.858, 0.875)	0.878	(0.872, 0.884)	.0012
Polynomial SVM	0.866	(0.858, 0.874)	0.866	(0.859, 0.871)	.3026
Logistic Regression	0.867	(0.859, 0.874)	0.876	(0.869, 0.883)	.0019
Linear SVM	0.871	(0.863, 0.878)	0.873	(0.865, 0.880)	.3383

acc—mean accuracy index for 30 runs.

a *p*‐value of one‐sided Wilcoxon–Mann–Whitney test. The maximal accuracy values are shown in bold.

We then compared the prediction accuracy of the inductive transfer learning method with that of SIFT. On the mouse dataset, SIFT has an accuracy of 84.9%, while the Random Forest classifier trained on the HumDiv dataset and used on the target feature space transformed using a neutral network has an accuracy of 88.4%.

We conclude that the application of transfer learning techniques can substantially improve the classification accuracy of deleterious mutations in nonhuman species.

### 3.3 Transfer learning for prediction of cattle data

Having learned how to best analyze mouse and dog data, we now move to the very important task of predicting deleterious mutations in farm animals. Indeed, as current cattle breeds have a very small effective population size (Pausch et al., 2015; Stachowicz, Sargolzaei, Miglior, & Schenkel, 2011) exacerbated by intense artificial selection (Bovine HapMap Consortium et al., 2009), cattle populations are susceptible to the increasing frequency of recessive deleterious alleles and, as a result, to the propagation of recessive disorders and homozygotes with a fatal phenotype (Pausch et al., 2015).

We found 1,892,964 mutations in 18,716 cattle proteins in the Ensembl database and classified them using transductive transfer learning based on reweighing samples according to their distance to human data. More precisely, we trained a Random Forest classifier on the HumVar data and reweighted the data according to the distances to cattle data. As a result, this method predicted 72% missense mutations to be deleterious and 28% to be neutral. The rate of deleterious mutations is higher compared to that obtained by SIFT: 54% deleterious and 46% neutral. According to Table 5, 90% of deleterious mutations predicted by SIFT are also predicted as deleterious by transfer learning, but only half of the neutral mutations predicted by SIFT are predicted as deleterious by transfer learning. This could be due to the high false‐negative rate (FNR) of SIFT (Chun & Fay, 2009; Di, Chan, Wei, Liu, & Zhou, 2009). It is possible that transfer learning predictions lead to high FPR, but this is unlikely given that that FNR and FPR were almost the same when the method was applied to classify dog and mouse data. The predictions for the cattle genomewide dataset are available in Table S4.

**Table 5:** Comparison of proportions of deleterious mutation predicted by transfer learning and SIFT in cattle genomewide dataset

TL predictions	SIFT predictions		
	Deleterious	Neutral	Total
Deleterious	49%	23%	72%
Neutral	5%	23%	28%
Total	54%	46%	100%

To verify the transfer learning predictions, we used the cattle62 dataset containing 31 neutral and 31 deleterious mutations (see Methods for the details). We correctly predicted 29 of 30 mutations found in the OMIA database as associated with different Mendelian diseases in cattle. For example, our method classified the W317R substitution in the Q2KIK0 protein as deleterious, which is known to compromise reproductive and rearing success in cattle (Pausch et al., 2015). Additionally, our method predicted that the L2153H substitution in B9X245 protein is disease‐causing and, in fact, it has been shown to cause hemophilia A in cattle (Khalaj et al., 2009). The list of all damaging mutations with annotations is available in Table S5. Given these results, our classifier may enable accurate predictions of deleterious mutations in whole‐genome analyses.

## 4 CONCLUSIONS

In this study, we have sought to demonstrate the potential of transfer learning as a set of methods for the mutation classification problem. One important advantage of transfer learning is that it provides a way to use known information about a problem of interest from one domain and apply it a new domain. Here, we have presented two methods of transferring knowledge from human mutation classification to classification of deleterious mutations in other species. In the first method, we used source data to train and tune classifier parameters where samples from the source data were weighted according to their distances to the target data. In the second method, we transformed the target data using a neural network trained on the source data. We developed classifiers that attain substantially better accuracy than programs currently used for classifying mutations. In the future, we will extend our approaches to classify the effects of deletions and insertions. Current large datasets also provide a chance to decipher epistasis effect. This will require learning from more distantly related species, such as yeast, which will be an interesting challenge.

## 5 DATA ARCHIVING STATEMENT

Data are available as supplementary information (Tables S1, S2, S3, S4 and S5). Implementation of transfer learning algorithm and Tables S2 and S4 are available at https://github.com/PlekhanovaElena/Transfer_learning.

## CONFLICT OF INTEREST

The authors declare no conflict of interests or competing financial interests.
