# Corrigendum

**DOI:** 10.1111/eva.12800

**Published:** 2019-11-01

The article by Plekhanova, Nuzhdin, Utkin, and Samsonova (2018) contains an error in the formula of weights of each sample from source data $x_{i}^{S}$ in the transductive transfer learning method. The correct text should read as:

Let $x^{S}$ be source data with *N* columns and *M* rows; $x^{T}$ be target data with *N* columns and *L* rows, *x_ij_* – an element from the *i*th row and *j*th column of data x; *m^s^*, *m^T^* – vectors, whose elements are the medians of the columns of $x^{S}$ and $x^{T}$, respectively. Then the weight of each sample from source data $x_{i}^{S}$ is

$$w_{i}=\exp\left\{-{\left[\frac{1}{L}\sum_{k=1}^{L}\sum_{j=1}^{N}{\left(x_{\mathrm{kj}}^{T}-x_{\mathrm{ij}}^{S^{\prime }}\right)}^{2}\right]}^{2}\right\},$$

where $x^{S^{\prime }}$ is source data shifted towards the target data median $x_{\mathrm{ij}}^{S^{{}^{\prime }}}=x_{\mathrm{ij}}^{S}-\left(m_{j}^{S}-m_{j}^{T}\right).$ Shifting towards the median accounts only for rotation of the source over the target data and not for the linear shift between them.

Additionally, in Table 3, the classification quality of mouse data for classifiers trained on HumVar without weights was presented incorrectly. The correct values are listed in Table 3 here.

**Table 3:** Comparative analysis of classification quality (accuracy on dog and mouse data) for classifiers, trained on source data with weights (+TL) and without weights (—). The maximal accuracy values achieved are shown in bold

	Dog				Mouse			
	Trained on HumDiv		Trained on HumVar		Trained on HumDiv		Trained on HumVar	
Classifier	+TL	—	+TL	—	+TL	—	+TL	—
Random Forest	0.855	0.638	**0.884**	0.879	**0.844**	0.679	0.841	0.828
Polynomial SVM	0.874	0.657	0.715	0.454	0.748	0.528	0.812	0.706
Gaussian SVM	0.686	0.662	0.754	0.618	0.613	0.560	0.833	0.674
Logistic Regression	**0.908**	0.667	0.855	0.700	0.772	0.565	**0.875**	0.851
Linear SVM	0.672	0.672	0.715	0.715	0.578	0.578	0.851	0.851

## References

[eva12800-bib-0001] Plekhanova, E. , Nuzhdin, S. V. , Utkin, L. V. , & Samsonova, M. G. (2018). Prediction of deleterious mutations in coding regions of mammals with transfer learning. Evolutionary Applications, 12(1), 18–28. 10.1111/eva.12607 30622632 PMC6304693

