# Supplementary material for: Prediction of deleterious mutations in coding regions of mammals with transfer learning
Source: Evol Appl. 2018 May 9;12(1):18–28. doi: 10.1111/eva.12607 (PMC6304693; doi:10.1111/eva.12607)
Supplement: Supplementary file 1 [file EVA-12-18-s001.pdf]

Supplementary information to "Prediction of deleterious mutations in coding regions of mammals with transfer learning"

Elena Plekhanova[1], Sergey V. Nuzhdin[2,1], Lev V. Utkin[1] and Maria G. Samsonova[1]

[1] Peter the Great St. Petersburg Polytechnic University, St. Petersburg, Russia
[2] Program Molecular and Computation Biology, Dornsife College of Letters, Arts, and Sciences, University of Southern California, Los Angeles, CA

Supplementary Table S1. List of deleterious and neutral mutations with known effect compiled for mouse, dog and cattle. For each mutation we present UniProtKB entry, position within the protein, type of amino-acid substitution, as well as the values of classification features described in Table 4. "mtype" column indicates whether mutation is neutral "0" or deleterious "1". (see Supplementary_table_S3.xlsx)

Supplementary Table S2. List of deleterious and neutral mutations with known effect compiled for the cattle genome-wide (CattleGW) dataset. For each mutation we present UniProtKB entry, position within the protein, type of amino-acid substitution, as well as the values of classification features described in Table 4.
(see Supplementary_table_S4.csv)

Supplementary Table S3. Optimal classification parameters for HumDiv and HumVar datasets.

Classifier	Parameters	HumDiv	HumVar
Random Forest	Number of estimators	1200	2500
Neural Network	Number of layers	(90, 20)	(30, 70, 10)
	Regularisation parameter	0.01	0.1
Polynomial SVM	Regularisation parameter	9000	6000
	Constant term	0.1	0.01
	Degree	3	3
Gaussian SVM	Regularisation parameter	18000	10000
Logistic Regression	Regularisation parameter	130	5
Linear SVM	Regularisation parameter	17	8
Boosted Gaussian NB	Number of estimators	3	3
	Boosting algorithm	SAMME.R	SAMME.R

For Naive Bayes (NB) and Deep Forest classifiers, default parameters were used.

Supplementary Table S4. Classification results in the cattle genome-wide dataset(see

Supplementary_table_S1.txt)

Supplementary Table S5. Information about deleterious mutations with known effect in the cattle62 dataset. For each mutation we present information about its UniProtKB entry, position within the protein, type of amino-acid substitution, as well as a link to the OMIA database entry and disease name.
(see Supplementary_table_S2.xlsx)

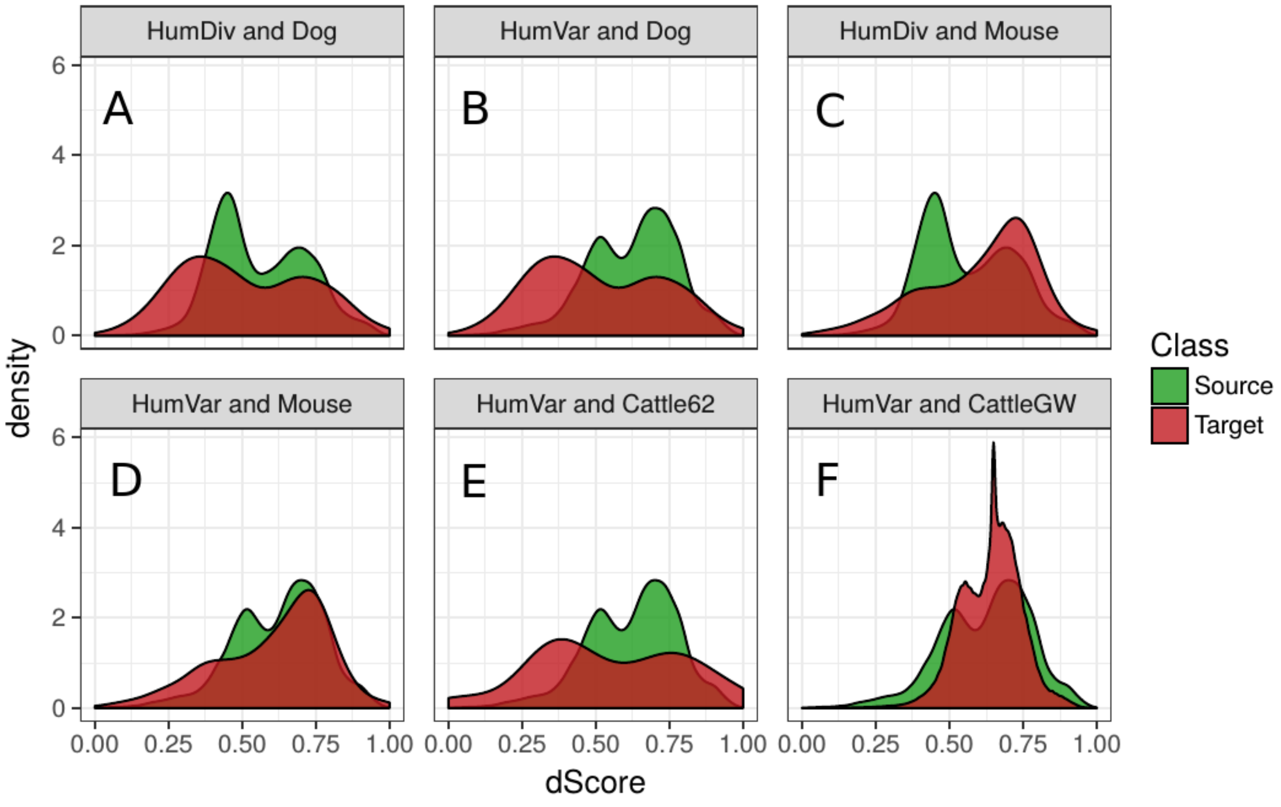

Supplementary Fig. S1. Distributions of normalised difference in PSIC score between mutant and wild alleles (dScore) in source and target datasets. A – HumDiv (source) and Dog (target), B- HumVar (source) and Dog (target), C- HumDiv (source) and Mouse (target), D – HumVar (source) and Mouse (target), E – HumVar (source) and Cattle62 (target), F - HumVar (source) and CattleGW (target). For each dataset in A – F there is apparent difference between distributions.

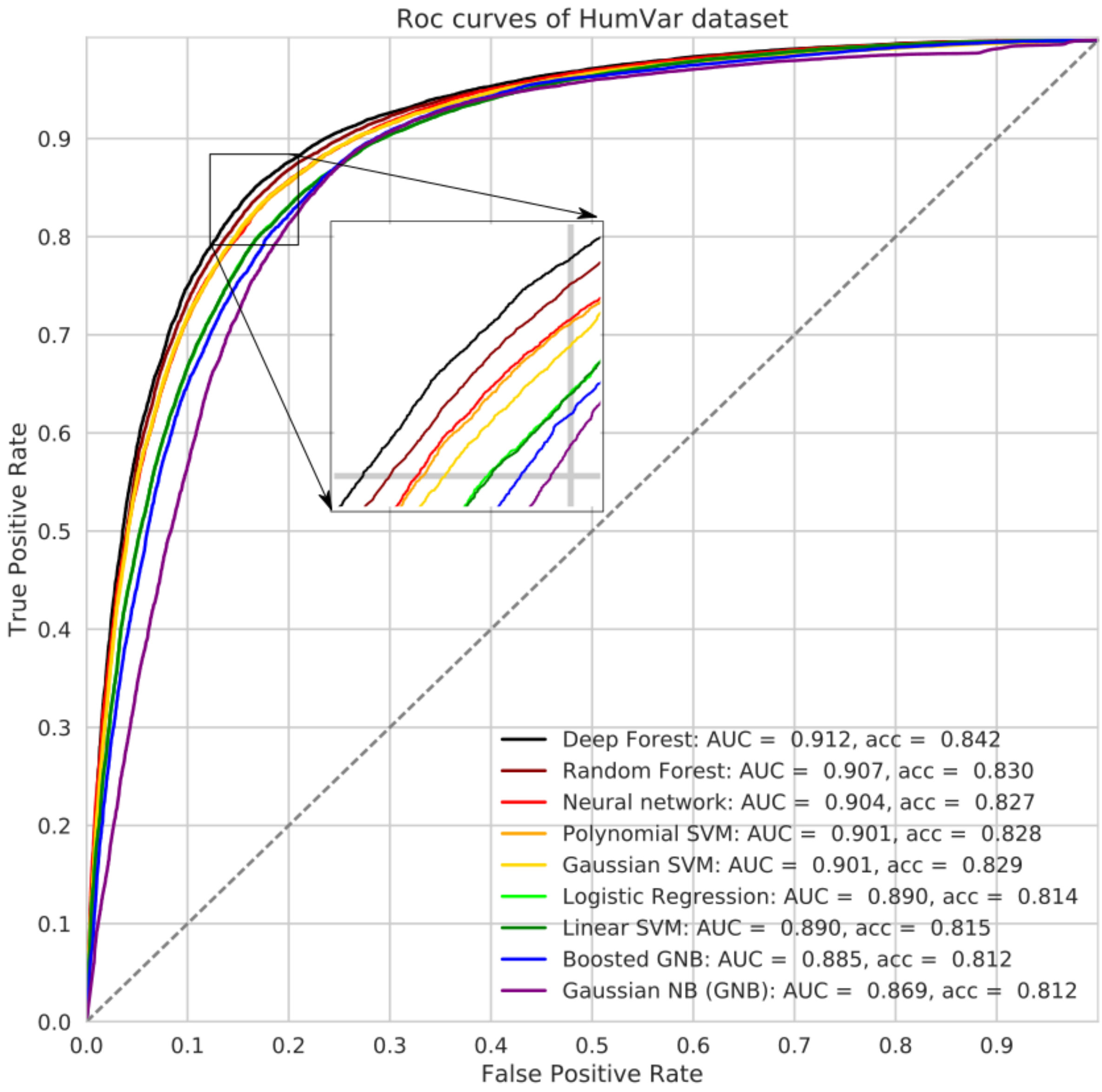

Supplementary Fig. S2. ROC-curves for different classifiers, trained on HumVar dataset. Values of quality metrics ordered by decreasing AUC values are shown adjacent to the classifier name. The dotted line corresponds to the ROC-curve for random guessing. The inset zooms in on the left upper quadrant to better distinguish the ROC-curves.
