# Supplementary material for: Prediction of deleterious mutations in coding regions of mammals with transfer learning
Source: Evol Appl. 2018 May 9;12(1):18–28. doi: 10.1111/eva.12607 (PMC6304693; doi:10.1111/eva.12607)
Supplement: Supplementary file 4 [file EVA-12-18-s004.html]

Supplementary\_table\_S4.zip (54651891)  
