# Supplementary material for: Prediction of deleterious mutations in coding regions of mammals with transfer learning
Source: Evol Appl. 2018 May 9;12(1):18–28. doi: 10.1111/eva.12607 (PMC6304693; doi:10.1111/eva.12607)
Supplement: Supplementary file 5 [file EVA-12-18-s005.zip › Transfer learning paper/Supplementary_info_final.docx]

# Supplementary information to "Prediction of deleterious mutations in coding regions of mammals with Transfer learning"

Elena Plekhanova^1^, Sergey V. Nuzhdin^2,1^, Lev V. Utkin^1^ and Maria G. Samsonova^1^

^1^ Peter the Great St. Petersburg Polytechnic University, St. Petersburg, Russia

^2^ Program Molecular and Computation Biology, Dornsife College of Letters, Arts, and Sciences, University of Southern California, Los Angeles, CA

**Supplementary Table S1. Classification results in the cattle genome-wide dataset.**

(see Supplementary_table_S1.txt)

**Supplementary Table S2. Information about deleterious mutations with known effect in the cattle62 dataset.** For each mutation we present information about its UniProtKB entry, position within the protein, type of amino-acid substitution, as well as a link to the OMIA database entry and disease name.
(see Supplementary_table_S2.xlsx)

**Supplementary Table S3. List of deleterious and neutral mutations with known effect compiled for mouse, dog and cattle.** For each mutation we present UniProtKB entry, position within the protein, type of amino-acid substitution, as well as the values of classification features described in Table 4. "mtype" column indicates whether mutation is neutral "0" or deleterious "1".
(see Supplementary_table_S3.xlsx)

**Supplementary Table S4. List of deleterious and neutral mutations with known effect compiled for the cattle genome-wide (CattleGW) dataset.** For each mutation we present UniProtKB entry, position within the protein, type of amino-acid substitution, as well as the values of classification features described in Table 4.
(see Supplementary_table_S4.csv)

**Supplementary Table S5. Optimal classification parameters for HumDiv and HumVar datasets.**

| **Classifier** | **Parameters** | **HumDiv** | **HumVar** |
| --- | --- | --- | --- |
| Random Forest | Number of estimators | 1200 | 2500 |
| Neural Network | Number of layers | (90, 20) | (30, 70, 10) |
|  | Regularisation parameter | 0.01 | 0.1 |
| Polynomial SVM | Regularisation parameter | 9000 | 6000 |
|  | Constant term | 0.1 | 0.01 |
|  | Degree | 3 | 3 |
| Gaussian SVM | Regularisation parameter | 18000 | 10000 |
| Logistic Regression | Regularisation parameter | 130 | 5 |
| Linear SVM | Regularisation parameter | 17 | 8 |
| Boosted Gaussian NB | Number of estimators | 3 | 3 |
|  | Boosting algorithm | SAMME.R | SAMME.R |

For Naive Bayes (NB) and Deep Forest, classifiers, define parameters were used.


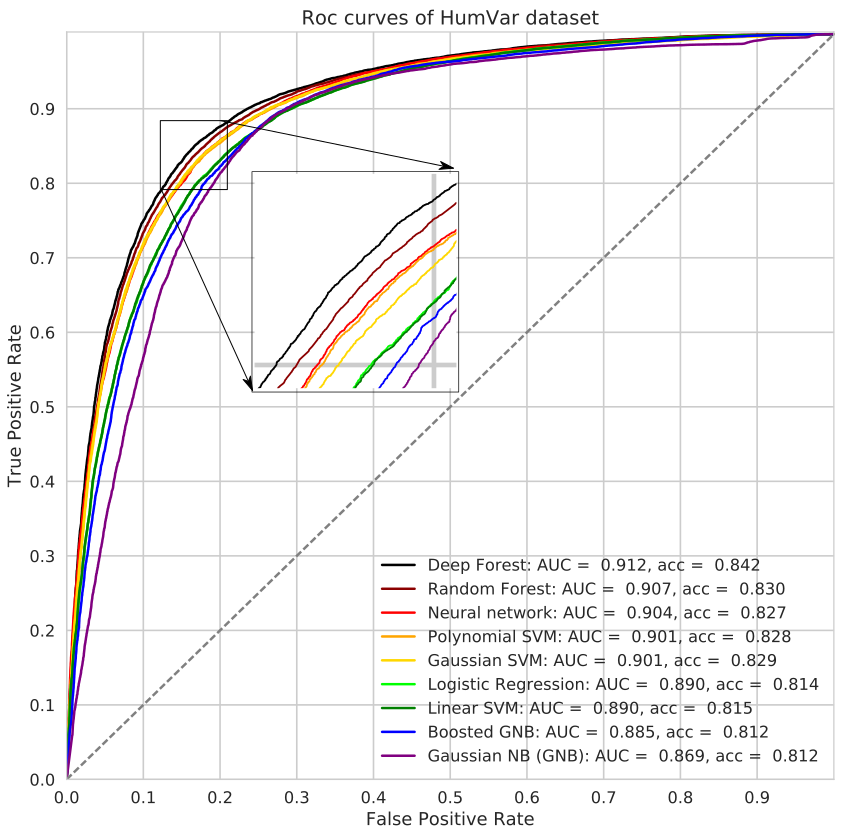


**Supplementary Fig. S1.** **ROC-curves for different classifiers, trained on HumVar dataset.** Values of quality metrics ordered by decreasing AUC values are shown adjacent to the classifier name. The dotted line corresponds to the ROC-curve for random guessing. The inset zooms in on the left upper quadrant to better distinguish the ROC-curves.


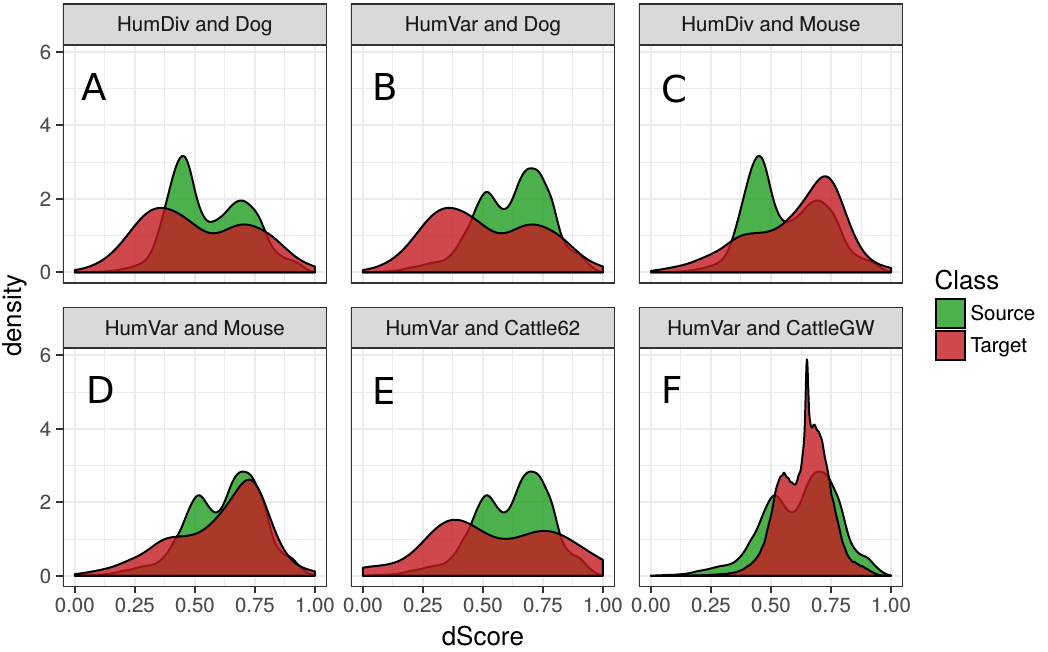


**Supplementary Fig. S2. Distributions of normalised difference in PSIC score between mutant and wild alleles (dScore) in source and target datasets.** A – HumDiv (source) and Dog (target), B- HumVar (source) and Dog (target), C- HumDiv (source) and Mouse (target), D – HumVar (source) and Mouse (target), E – HumVar (source) and Cattle62 (target), F - HumVar (source) and CattleGW (target). For each dataset in A – F there is apparent difference between distributions.
