# Supplementary material for: Prediction of deleterious mutations in coding regions of mammals with transfer learning
Source: Evol Appl. 2018 May 9;12(1):18–28. doi: 10.1111/eva.12607 (PMC6304693; doi:10.1111/eva.12607)
Supplement: Supplementary file 5 [file EVA-12-18-s005.zip › Transfer learning paper/TL_paper_final_190118_clean.docx]

Prediction of deleterious mutations in coding regions of mammals with Transfer learning

Elena Plekhanova^1^, Sergey V. Nuzhdin^2,1^, Lev V. Utkin^1^ and Maria G. Samsonova^1^

^1^ Peter the Great St. Petersburg Polytechnic University, St. Petersburg, Russia

^2^ Program Molecular and Computation Biology, Dornsife College of Letters, Arts, and Sciences, University of Southern California, Los Angeles, CA

E-mail adresses: [plekhanova-elena@yandex.ru](mailto:plekhanova-elena@yandex.ru) (Elena Plekhanova), [snuzhdin@usc.edu](mailto:snuzhdin@usc.edu) (Sergey V. Nuzhdin), [lev.utkin@mail.ru](mailto:lev.utkin@mail.ru) (Lev V. Utkin) and [m.samsonova@spbstu.ru](mailto:m.samsonova@spbstu.ru) (Maria G. Samsonova)

Running head: Damaging variants detection with Transfer Learning

## **Abstract**

The genomes of mammals contain thousands of deleterious mutations. It is important to be able to recognize them with high precision. In conservation biology, the small size of fragmented populations results in accumulation of damaging variants. Preserving animals with less damaged genomes could optimize conservation efforts. In breeding of farm animals, trade-offs between farm performance versus general fitness might be better avoided if deleterious mutations are well classified. In humans, the problem of such a precise classification has been successfully solved, in large part due to large databases of disease-causing mutations. However, this kind of information is very limited for other mammals. Here, we propose to better use information available on human mutations to enable classification of damaging mutations in other mammalian species. Specifically, we apply Transfer learning - machine learning methods, improving small data set for solving a focal problem (recognizing damaging mutations in our companion and farm animals) due to use of much large data sets available for solving a related problem (recognizing damaging mutations in humans). We validate our tools using mouse and dog annotated datasets and obtain significantly better results in companion to the SIFT classifier. Then we apply them to predict deleterious mutations in cattle genome-wide dataset.

**Keywords**: deleterious mutations, Transfer learning, classification

**Article type:** Special issue Original Research article

## **Introduction**

The amount of data gathered in biological experiments grows at an alarming rate, especially in the field of genomics. However, the field of knowledge as a whole remains fractured, which is an acute problem. Consequently, one of the questions of comparative genomics is: when we have a few well-studied model organisms, how can we transfer this knowledge to phylogenetically related organisms? Indeed, cross-referencing the information from connected fields of science and art is a common practice. For a real-life example, consider a person who plays the violin, and desires to learn the piano. It would be easier for them to learn to play piano than for a person without any experience with a musical instrument. Similar transfers of knowledge are sometimes used to solve classification and regression problems. This process is formalized in machine learning techniques called ‘Transfer learning’.

Here we consider the application of Transfer learning to the problem of classification of mutations into categories ‘deleterious’ versus ‘neutral’. This problem is interesting for both theoretical and practical considerations. Not only can we use this knowledge to explore functional genome variation in multiple species, but we can also study their evolution, ultimately with an aim to understand why a mutant allele classified as deleterious in one species may be in fact a normal allele in another species (Charlesworth, 2012; Hartfield, Glémin, Yu, & Purugganan, 2014; Kaiser & Charlesworth, 2009). Further, enhanced understanding of deleterious genomic content may enhance selection and breeding in crop species, and may also improve veterinary medicine capabilities in species such as cattle, pigs, and dogs. For example, Raszek et al. (Raszek, Guan, & Plastow, 2016) describes the application of comparative genomic analysis for treatment of infectious disease and understanding of developmental abnormality, which is a major economic burden in cattle production worldwide. Similarly, Knol et al. (Knol, Nielsen, & Knap, 2016) considers the application of functional, knowledge-enabled genomic selection in commercial pig breeding.

Here, we utilize the transfer of knowledge from the classification of human mutations to similar categorizations in other mammalian species. This direction of knowledge transfer was chosen because of the abundance of both information about the properties of human mutations (Kondrashov, 2012; Capriotti, Calabrese, & Casadio, 2006) and inferences from association studies (MacArthur et al., 2017), and relatively small amount of information of mutations in other species. Firstly, we train a classifier on a labeled human data. Further, we use a relatively small amount of information from other species to reduce any biases due to human-centric training when applied to other mammals, particularly to mouse, dog, and cattle.

Formally, we consider deleterious mutations as a genetic alteration that increases an individual's susceptibility or predisposition to a certain disease or disorder. Nearly all deleterious mutations in non-human mammals are found in the coding part of the genome and are typically missense mutations, i.e. those which cause amino-acid changes in the corresponding protein. However, many missense mutations do not cause a disease (Huber, Kim, Marsden, & Lohmueller, 2017; Kim, Huber, & Lohmueller, 2017). Here, we classify mutations into one of two classes: ‘deleterious’ or ‘neutral’. To accomplish this task, there are a number of characteristics that need to be known about the mutation, such as whether it is a transition or a transversion (Stoltzfus et al., 2016), and the frequency of a particular mutation in the population (a detailed description of these characteristics is available in the Methods section). Thus, for each mutation, there are a number of features for classification.

There are two main ways to perform classification. Firstly, the values of the features can be compared with some threshold. For example, this concept was implemented in SIFT (Sim et al., 2012), and PolyPhen1 (Ramensky, Bork, & Sunyaev, 2002). SIFT, in particular, uses the conservation score of the sequence as the threshold. Secondly, classification can be performed by using machine learning techniques. In this case, the set of labeled mutations is used as a training set for a classifier, and then the trained classifier is applied to make predictions on an unlabeled set. The advantage of machine learning methods is that parameters do not have to be manually set, but can instead be identified automatically based on the labeled set. This concept was realized in PolyPhen-2 (Adzhubei et al., 2010), where the classification consists of two steps. The first step is feature preparation: the software collects necessary information about a mutation using sequence characteristics, multiple alignment scores, and information about the 3D structure of the resulting protein. The second step is classification by applying a naïve Bayes approach. In this work, we use PolyPhen-2’s preparation step, however the second step, the classification of data in a species of interest, was optimized.

A substantial amount of research has been devoted to classification of human mutations (Pabinger et al., 2014). However only two well-known programs are useful for classification of mutations in non-human mammals, SIFT (Sim et al., 2012) and MAPP (Stone & Sidow, 2005). They both are based on evolutionary conservation of polymorphism inferred through multiple alignment scores (MAPP also uses a phylogenetic tree) to make a prediction. While their predictions are reasonably accurate, both SIFT and MAPP might potentially be improved by taking advantage of additional classification features, such as the 3D structure of the resulting protein, or properties of already classified mutations. Further, their performance might be enhanced with incorporation of machine learning techniques, for instance choosing the thresholds automatically, as PolyPhen-2 does for classification of human mutations.

There is, in principle, an option in PolyPhen-2 for classification of mutations in non-human species. However, it is an experimental feature and it makes predictions using a classifier trained on human data. In our work, we aim to modify this procedure and use transfer learning techniques in order to improve the accuracy of the classification via re-training on species-specific, though limited, data sets.

An assumption of traditional machine learning is that the training data and testing data are taken from the same domain, such that the input feature space and data distribution characteristics are the same. However, in some real-world machine learning scenarios, this assumption is unfeasible because training data is expensive or difficult to collect (Weiss, Khoshgoftaar, & Wang, 2016). Therefore, there is a need to create high-performance learners trained with more easily obtained data from different domains. The methodology addressing this need is referred to as Тransfer learning.

### The transfer learning problem can be formally defined as follows. Let domain
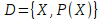
 be characterized by two parts: a feature space
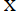
 and a marginal probability distribution
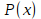
. For a given domain
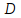
, a task
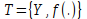
 is defined by two parts: a label space
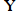
, and a predictive function
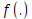
, which is learned from labeled features. Now,
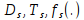
 are referred to as the source dataset, source task and source predictive function, respectively, and
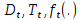
 are referred to as the target dataset, target task and target predictive function, respectively. Transfer learning is the set of machine learning methods that aim to improve the target predictive function
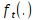
 by using related information from the source dataset
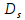
 and source task
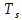
, where
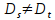
 or
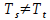
.

Transfer learning methods are actively used for a large variety of real-world tasks from atmospheric dust aerosol particle classification to face motion recognition (Ma, Gong, & Mao, 2015; Perlich, Dalessandro, Stitelman, Raeder, & Provost, 2013; Kan, Wu, Shan, & Chen, 2014). There are several applications of transfer learning methods in bioinformatics, for example, for recognition of splicing sites (Schweikert, Schweikert, & Widmer, 2008; Widmer, Leiva, Altun, & Rätsch, 2010), gene expression analysis (Chen & Huang, 2010; Q. Xu, Xue, & Yang, 2011), and image recognition (Bi, Xiong, Yu, Dundar, & Rao, 2008). However, despite a growing amount of SNP data, only a few studies have applied transfer learning to SNP-related analyses (Chen & Huang, 2010; Puniyani, Kim, & Xing, 2010; Q. Xu et al., 2011).

Different techniques of transfer learning are best used depending on the research needs (Weiss et al., 2016). We want to use both the labeled source dataset of human mutations and the small labeled target datasets of mouse, dog, or cattle mutations to better predict unlabeled mutations in these non-human species. So, we assume that source data is labeled and target data is unlabeled or partly labeled. Under these circumstances, one should use Transductive and Inductive transfer learning techniques.

Transductive transfer learning is an example of Instance transfer based on reweighting samples of source data according to their distances to target data. Chattopadhyay et al. (Chattopadhyay et al., 2012) proposed to use this approach while working with multiple labeled source domains. The main idea is to use a combination of source domain classifiers, with weights assigned as a function of the closeness in conditional distribution between each source and target domain, to label the unlabeled target data. Other authors have used different sample weights metrics to account for instances from one source domain (Y. Xu et al., 2017).

The Inductive transfer learning solution belongs to feature-based Transfer learning approaches. It transforms the feature spaces of source and target data to make them more similar and, thus, to decrease classification error. The transformation of source data to a new feature space can be implemented with a denoising autoencoder (Glorot, Bordes, & Bengio, 2011), or convolutional neural networks (Oquab, Bottou, Laptev, & Sivic, 2014).

Here, we firstly demonstrate the applicability of Transductive and Inductive transfer learning techniques for the prediction of deleterious mutations in dog and mouse datasets, and then apply Transductive transfer learning to predict deleterious mutations in cattle.

**Results and Discussion**

To obtain reasonable predictions for the target data, we need to ascertain that a classifier attains significant accuracy on the source data. Therefore, we compared the performance of different classification methods on human data and selected the best classifiers that were further used in transfer learning tasks.

### **Classification of human data**

The human data consists of two datasets, HumDiv and HumVar. The HumDiv dataset (Ramensky et al., 2002) consists of 13103 mutations that are either neutral with respect to closely related mammalian homologs or cause human Mendelian diseases. HumVar (Capriotti, Calabrese, & Casadio, 2006), consisting of 43,347 mutations, treats any disease-causing mutation as damaging and it assumes that common human non-synonymous SNPs (MAF>1%) are neutral, as long as they have not been annotated as disease-causing.

We first tested the performance of 9 different classifiers: Deep and Random Forest, Neural Network, Gaussian, Polynomal and Linear SVM, Logistic Regression, Gaussian Naïve Bayes and Boosted Gaussian Naïve Bayes. The quality of each predictor was assessed using two metrics: AUC, which is the area under the ROC curve, and the accuracy rate, which is the fraction of correct predictions. On both datasets, the best results were achieved with Deep Forest, Random Forest, Neural Network, Polynomial SVM, Gaussian SVM, Linear SVM, and Logistic regression (Fig. 1, Supplementary Fig. S1). On the HumDiv dataset, the classification error varies from 5 to 7% and AUC values range from 98 to 99%. On the HumVar dataset, the classification errors were larger ranging from 16 to 18% and the AUC value varied from 89 to 91%. As a result, we keep these classifiers for further analysis.

**Fig. 1. ROC-curves for different classifiers, trained on HumDiv data.** Values of quality metrics ordered by decreasing AUC values are shown adjacent to the classifier name. The dotted line corresponds to the ROC-curve for random guessing. The inset zooms in on the left upper quadrant to better distinguish the ROC-curves.

Next, we compared the classifiers with the Naive Bayes classifier used by PolyPhen-2 (Adzhubei et al., 2010) and with the SIFT classifier (Sim et al., 2012). Most classifiers better predicted deleterious mutations than PolyPhen-2 given that they resulted in larger values of TPR (True Positive Rate) for a given FPR (False Positive Rate) than PolyPhen-2 (Table 1). Note, that the Deep Forest classifier showed the best results, which were significantly better than PolyPhen-2 predictions. As for the SIFT classifier, it shows results worse than Polyphen-2: 0.863 accuracy on HumDiv and 0.796 on HumVar dataset.

**Table 1. Comparison of classifier performance on human datasets.**

|  | **HumDiv** | | | | **HumVar** | | | |
| --- | --- | --- | --- | --- | --- | --- | --- | --- |
| **Classifier** | **FPR** | | | **acc** | **FPR** | | | **acc** |
|  | 0.05 | 0.10 | 0.20 |  | 0.05 | 0.10 | 0.20 |  |
|  | **TPR** | | |  | **TPR** | | |  |
| Deep Forest | **0.950** | **0.986** | 0.996 | **0.951** | **0.584** | **0.749** | **0.876** | **0.842** |
| Random Forest | 0.947 | **0.986** | **0.997** | 0.940 | 0.563 | 0.733 | 0.868 | 0.830 |
| Neural Network | 0.916 | 0.972 | 0.993 | 0.939 | 0.548 | 0.716 | 0.857 | 0.827 |
| Gaussian SVM | 0.916 | 0.975 | 0.995 | 0.940 | 0.551 | 0.719 | 0.857 | 0.829 |
| Polynomial SVM | 0.917 | 0.973 | 0.995 | 0.940 | 0.549 | 0.716 | 0.854 | 0.828 |
| Logistic Regression | 0.895 | 0.961 | 0.992 | 0.931 | 0.484 | 0.666 | 0.831 | 0.814 |
| Linear SVM | 0.897 | 0.961 | 0.992 | 0.933 | 0.483 | 0.667 | 0.831 | 0.815 |
| Boosted Gaussian NB | 0.850 | 0.936 | 0.977 | 0.805 | 0.445 | 0.650 | 0.822 | 0.812 |
| Gaussian NB | 0.794 | 0.928 | 0.978 | 0.805 | 0.341 | 0.568 | 0.813 | 0.812 |
| PolyPhen-2 | 0.78 | 0.89 | 0.96 | 0.89 | 0.53 | 0.68 | 0.83 | 0.81 |

1. TPRs (True Positive Rates) corresponding to a given FPR (False Positive Rates) are provided. The values of the accuracy metric (acc) are given for all classifiers ordered by decreasing of AUC metric (see Fig. 1, Supplementary Fig. S2). Cells with AUC or acc values no less than corresponding PolyPhen-2 values are filled in yellow while cells with AUC or acc values smaller than corresponding PolyPhen-2 values are colored light blue. In each column, the maximal value is in bold.

### **Transfer learning for classification of mouse and dog data**

Due to the relatively small amount of information on deleterious mutations, the application of traditional machine learning methods to mutation classification in non-human species does not seem possible. A good alternative may be to use transfer learning trained with more easily obtained human. Here, we tested the applicability of two transfer learning methods, namely Transductive and Inductive transfer learning, to predict deleterious mutations in dog and mice.

The Transfer learning methods improve the target data classification by using related information from other source datasets. We aim to transfer knowledge from classification of human mutations to similar tasks in dog and mouse and, consequently, we used HumVar and HumDiv as source datasets.

To construct the target dog and mouse datasets, we used UniprotKB (Consortium, 2017) and either OMIA (Lenffer et al., 2006) or MGI (Eppig et al., 2017) databases (see Methods for a more detailed description). Mutations associated with Mendelian diseases are considered damaging. Putative neutral mutations were sampled either from positions that differ in alignments between human proteins and their closely related mammalian homologs or from common variants segregating within species.

***Transductive transfer learning***

The Transductive transfer learning assumes that target data is unlabeled. We applied Instance transfer learning to reweight samples from the source dataset according to their distance to the target data. To assess the classification results, we trained each classifier independently on the source data with and without weights and then tested its performance on the target data. For this analysis we considered only five classifiers, for which reweighing is possible: Random Forest, Polynomial SVM, Gaussian SVM, Logistic Regression and Linear SVM.

**Table 2. Comparison of the quality of deleterious mutations classification in dog and mouse datasets for classifiers, trained with weights (+TL) and without weights (**—**) on source data.**

| **Classifier** | **Dog** | | | | **Mouse** | | | | | |
| --- | --- | --- | --- | --- | --- | --- | --- | --- | --- | --- |
|  | Trained on **HumDiv** | | Trained on **HumVar** | | Trained on **HumDiv** | | | Trained on **HumVar** | | |
|  | **+TL** | — | **+TL** | — | | **+TL** | — | | **+TL** | — |
| Random Forest | 0.855 | 0.638 | **0.889** | 0.884 | | **0.846** | 0.682 | | 0.841 | 0.682 |
| Polynomial SVM | 0.874 | 0.657 | 0.715 | 0.454 | | 0.764 | 0.764 | | 0.812 | 0.528 |
| Gaussian SVM | 0.686 | 0.662 | 0.753 | 0.618 | | 0.655 | 0.539 | | 0.833 | 0.560 |
| Logistic Regression | **0.908** | 0.667 | 0.855 | 0.701 | | 0.777 | 0.576 | | **0.875** | 0.565 |
| Linear SVM | 0.672 | 0.672 | 0.715 | 0.715 | | 0.597 | 0.597 | | 0.852 | 0.568 |

In each cell, we present the accuracy rate reached by a classifier. The maximal accuracy values achieved are shown in bold.

As evident from Table 2, the application of Transductive transfer learning does not impair the performance of any classifier and, furthermore, improves the performance of several of them. The best results were reached with Random Forest and Logistic regression. Improvement was most noticeable in the following source-target data pairs: a) HumDiv-dog data, where classification using Logistic Regression was improved by 23,6% in comparison to the best result without using Transfer learning; b) HumVar-mouse data, where improvement of classification with the same classifier is 19,3% in comparison to the best result without using Transfer learning.

Even though it is the most often used tool to predict deleterious mutations in non-human species, we showed that SIFT is significantly less accurate in predicting deleterious mutations in dog and mouse datasets than Transfer learning methods. On the dog dataset, SIFT achieves an accuracy of 85.2% while Logistic regression trained on the HumDiv dataset has an accuracy of of 90.8%. On the mouse dataset, SIFT has an accuracy of 84.9% while the Logistic regression classifier trained on the HumVar dataset has an accuracy of 87.5%.

***Inductive transfer learning***

Inductive transfer learning assumes that the target data is labeled and aims to improve classification of the target data by transforming the feature spaces of both source and target data to make them more similar. Here we transform the target feature space using a neural network trained on source data. To estimate classification accuracy, each classifier was independently trained either on one third of the transformed or initial target data and then tested on the remaining part of the target data. To test this approach, we used the HumVar and mouse datasets as source and target data, respectively (see Methods for the details).

**Table 3. Comparative analysis of classification quality (accuracy) for classifiers trained without transformation (in initial feature space) and with transformation (in transformed space from the use of a neutral network) on mouse data.**

| **Classifier** | **Without transformation** | | **Without transformation** | | **p-value**** | |
| --- | --- | --- | --- | --- | --- | --- |
|  | **acc*** | **Conf. interval** | **acc** | **Conf. interval** | |  |
| Deep Forest | **0.879** | (0.873, 0.884) | 0.881 | (0.877, 0.887) | | 0.160 |
| Random Forest | 0.875 | (0.869, 0.882) | **0.884** | (0.879, 0.889) | | 0.0014 |
| Neural Network | 0.840 | (0.830, 0.850) | 0.866 | (0.854, 0.873) | | 0.0005 |
| Gaussian SVM | 0.866 | (0.858, 0.875) | 0.878 | (0.872, 0.884) | | 0.0012 |
| Polynomial SVM | 0.866 | (0.858, 0.874) | 0.866 | (0.859, 0.871) | | 0.3026 |
| Logistic Regression | 0.867 | (0.859, 0.874) | 0.876 | (0.869, 0.883) | | 0.0019 |
| Linear SVM | 0.871 | (0.863, 0.878) | 0.873 | (0.865, 0.880 | | 0.3383 |

*acc - mean accuracy index for 30 runs, **p-value of one-sided Wilcoxon-Mann-Whitney test. The maximal accuracy values are shown in bold.

As it is evident from Table 3, the classifiers can reach on average 87.9% accuracy in the initial feature space and 88.4% accuracy in the transformed one. For Random Forest, Neural Network, Gaussian SVM and Logistic Regression, the classification in transformed feature space leads to more accurate predictions according to the Wilcoxon-Mann-Whitney test (P<0.0025) (Wilcoxon, 1945).

We then compared the prediction accuracy of the Inductive transfer learning method with that of SIFT. On the mouse dataset, SIFT has an accuracy of 84.9% while the Random Forest classifier trained on the HumDiv dataset and used on the target feature space transformed using a neutral network has an accuracy of 88.4%.

We conclude that the application of Transfer learning techniques can substantially improve the classification accuracy of deleterious mutations in non-human species.

**Transfer learning for prediction of cattle data**

Having learned how to best analyze mouse and dog data, we now move to the very important task of predicting deleterious mutations in farm animals. Indeed, as current cattle breeds have a very small effective population size (Pausch et al., 2015; Stachowicz, Sargolzaei, Miglior, & Schenkel, 2011) exacerbated by intense artificial selection (Bovine HapMap Consortium et al., 2009), cattle populations are susceptible to the increasing frequency of recessive deleterious alleles and, as a result, to the propagation of recessive disorders and homozygotes with a fatal phenotype (Pausch et al., 2015).

We found 1,892,964 mutations in 18,716 cattle proteins in the Ensembl database and classified them using Transductive transfer learning based on reweighing samples according to their distance to human data. More precisely, we trained a Random Forest classifier on the HumVar data and reweighted the data according to the distances to cattle data. As a result, this method predicted 72% missense mutations to be deleterious and 28% to be neutral. The rate of deleterious mutations is higher compared to that obtained by SIFT: 54% deleterious and 46% neutral. According to Table 4, 90% of deleterious mutations predicted by SIFT are also predicted as deleterious by Transfer learning, but only half of the neutral mutations predicted by SIFT are predicted as deleterious by transfer learning. This could be due to the high false-negative rate (FNR) of SIFT (Chun & Fay, 2009; Di, Chan, Wei, Liu, & Zhou, 2009). It is possible that Transfer learning predictions lead to high FPR, but this is unlikely given that that FNR and FPR were almost the same when the method was applied to classify dog and mouse data. The predictions for the cattle genome-wide dataset are available in Supplementary table S1.

Table 4. Comparison of proportions of deleterious mutation predicted by Transfer learning and SIFT in cattle genome-wide dataset.

|  | **SIFT predictions** | | |
| --- | --- | --- | --- |
| **TL predictions** | deleterious | neutral | total |
| deleterious | 49% | 23% | 72% |
| neutral | 5% | 23% | 28% |
| total | 54% | 46% | 100% |

In order to verify the transfer learning predictions, we used the cattle62 dataset containing 31 neutral and 31 deleterious mutations (see Methods for the details). We correctly predicted 29 out of 30 mutations found in the OMIA database as associated with different Mendelian diseases in cattle. For example, our method classified the W317R substitution in the Q2KIK0 protein as deleterious, which is known to compromise reproductive and rearing success in cattle (Pausch et al., 2015). Additionally, our method predicted that the L2153H substitution in B9X245 protein is disease-causing and, in fact, it has been shown to cause Hemophilia A in cattle (Khalaj et al., 2009). The list of all damaging mutations with annotations is available in Supplementary Table S2. Given these results, our classifier may enable accurate predictions of deleterious mutations in whole genome analyses.

### **Conclusions**

In this paper, we have sought to demonstrate the potential of Transfer learning as a set of methods for the mutation classification problem. One important advantage of Transfer learning is that it provides a way to use known information about a problem of interest from one domain and apply it a new domain. Here we have presented two methods of transferring knowledge from human mutation classification to classification of deleterious mutations in other species. In the first method, we used source data to train and tune classifier parameters where samples from the source data were weighted according to their distances to the target data. In the second method, we transformed the target data using a neural network trained on the source data. We developed classifiers that attain substantially better accuracy than programs currently used for classifying mutations. In the future, we will extend our approaches to classify the effects of deletions and insertions. Current large data sets also provide a chance to decipher epistasis effect. This will require learning from more distantly related species, such as yeast, which will be an interesting challenge.

## **Material and Methods**

### **Datasets**

To apply machine learning technique to the problem of mutation classification, we needed to create labeled (deleterious versus neutral) datasets of mutations in order to train classifiers and assess their performance. Sets of deleterious mutations are usually compiled from databases with a focus on disease-causing mutations. Sets of neutral mutations are composed of non-synonymous single-nucleotide mutations that satisfy one of two criteria: they have been fixed during divergence between the species of interest and its closely related species or their MAF in population is at least 5% (Ng & Henikoff, 2001). More dеtailed information on the datasets used as well as on how they are created is presented below.

***Human datasets***

We used the two standard human datasets HumDiv and HumVar that are applied by PolyPhen-2 to train our classifier. The HumDiv dataset (Adzhubei et al., 2010) contains 5564 mutations from the UniProtKB database that are known to cause Mendelian diseases and a set of 7539 DNA variants between human proteins and their closely related mammalian homologs that are assumed to be non-damaging. The HumVar dataset (Capriotti et al., 2006) consists of 22196 human disease-causing mutations from UniProtKB and 21151 neutral mutations that are common human nsSNPs (MAF>1%) without annotated involvement in disease. Note, that the HumDiv dataset contains annotated mutations directly associated with human diseases, while the HumVar dataset is more noisy since its neutral mutation subset includes many mildly deleterious alleles.

***Mouse dataset***

We used both the MGI database (Eppig et al., 2017) and the Disease Ontology Database (Kibbe et al., 2015) to retrieve a set of 189 amino acid substitutions associated with different mouse diseases.

A set of 188 neutral mutations was compiled from non-synonymous SNPs present in 28 strains of mouse. We selected positions that were annotated in more than 8 *Mus* strains and that were different in no less than 2 strains. Thus, in total the data set consists of 377 mouse mutations of which 189 are deleterious and 188 are neutral (see Supplementary Table S3).

***Dog and cattle62 datasets***

The dog and cattle (called cattle62) datasets were generated similarly. To retrieve deleterious mutations, we used the OMIA database (Online Mendelian Inheritance in Animals) (Lenffer et al., 2006) containing information on deleterious mutations in animals. We considered only Mendelian diseases with known key missense mutation.

The sets of neutral mutations were generated from non-synonymous single-nucleotide mutations in homologous proteins using the UniProtKB database (Consortium, 2017). From this database, only the entries that were reviewed by database curators were selected. Then, for each entry, the following procedure was applied:

1. From a set of sequences similar to a query protein and retrieved with BLASTp (Altschul et al., 1997), only mammalian proteins with more than 95% identity to the input sequence were selected.
2. Clustal Omega (Sievers et al., 2014) was used to construct multiple alignments for sequences selected in the previous step.
3. The only amino-acid substitutions considered were those that were isolated (i.e. not present in a continuous block of substituted residues) and independent (i.e. there were no other substitutions in the same sequences of alignment). This helps us to avoid the phenomenon of correlated mutational behavior between columns of a multiple sequence alignment (Kowarsch, Fuchs, Frishman, & Pagel, 2010). In addition, we chose only substitutions present in no less than 2 aligned sequences.

The generated dog dataset consists of 207 mutations of which 103 are deleterious and 104 are neutral. For cattle62, we retrieved a set of 62 mutations of which 30 were deleterious and 32 neutral (see Supplementary Table S3).

.

***CattleGW dataset***

The cattle genome-wide (CattleGW) dataset was created using the Ensembl database. We found approximately 100,000,000 different genetic variants in cattle, almost 2,000,000 of which were missense mutations in 21,599 transcripts corresponding to 21,370 proteins in the UniprotKB database. For each mutation, we collected information about its position within the protein, type of amino-acid substitution, and SIFT prediction score. We used Polyphen-2 to prepare features for further classification of 1892964 mutations in 18716 proteins (see Supplementary Table S4).

**Feature description**

To make predictions about the effects of non-synonymous SNPs on protein function, we need to characterize these substitutions with informative predictive features. We used 14 predictive features (Table 5) of which 11 are standard predictive features used by the PolyPhen-2 algorithm (Adzhubei et al., 2010). Three additional features were added to improve final classification: Grantham score, which predicts evolutionary distance between two amino acids (Grantham, 1974); BLOSUM62 substitution score; (Henikoff & Henikoff, 1992); and PDB_id, which is an indicator of the availability of 3D structure information.

In all of the datasets, all predictive feature values were centered and normalized. Missing values were imputed with median values.

Table 5. Description of features used in classification.

| **Feature name** | **Description*** |
| --- | --- |
| GScore | Grantham score |
| BScore | amino acid score in BLOSUM62 matrix |
| Score1 | PSIC score (Sunyaev et al., 1999) for wild type amino acid residue (before substitution) |
| dScore | difference of PSIC (Sunyaev et al., 1999) scores for two amino acid residue variants (before and after substitution) |
| Nobs | number of residues observed at the substitution position in multiple alignment (without gaps) |
| NormASA | normalized accessible protein surface area |
| dVol | change in residue side chain volume |
| dProp | change in solvent accessible surface propensity resulting from the substitution |
| B.fact | normalized B-factor (temperature factor, (Chasman & Adams, 2001) for the residue |
| IdPmax | maximum congruency of the mutant amino acid residue to all sequences in multiple alignment |
| IdQmin | query sequence identity with the closest homologue deviating from the wild type amino acid residue |
| PDB_id | availability of PDB (Berman et al., 2000) protein structure identifier |
| Transv | substitution type (transversion or transition) |
| PfamHit | Availability Pfam (Finn et al., 2016) protein structure identifier |

*More detailed description is available at <http://genetics.bwh.harvard.edu/pph2/dokuwiki/appendix_a>.

### **The classifiers**

### We used the following set of different classifiers, which includes probabilistic ones: Naive Bayes (NB) with Gaussian kernel, Boosted Naive Bayes classifier (Freund & Schapire, 1999), Logistic Regression, Support Vector Machines (SVM) (Cortes & Vapnik, 1995) with three different kernels (linear, Gaussian (rbf), polynomial), Random Forest (Breiman, 2001), Neural Network (Jantzen, 1998) and a relatively new approach called Deep Forest (Zhou & Feng, 2017). We used two performance measures: AUC, which is the area under a ROC-curve, and accuracy (acc), which is the proportion of correctly classified samples. The classes were balanced so that the accuracy metrics worked correctly.

We trained and tuned the classifiers on the HumDiv and HumVar datasets. To avoid overfitting, 5-fold cross-validation was performed. Specifically, a dataset was split into 5 approximately equal parts (or folds) of which four parts were used for training and the fifth part for validation. This procedure was repeated 5 times with different parts used for validation each time. The performance measure is an average of the values computed at each iteration. While the dataset is usually split into folds randomly, we created folds such that all mutations in the same protein fell into the same fold. This was done in order to avoid overfitting in the situation where we train and test a classifier on the same protein.

The grid search method guided by the accuracy metric was used to tune classifiers (see best parameters in Supplementary Table S5).

To construct classifiers, we used the GaussianNB, LogisticRegression, LinearSVC, SVC, RandomForestClassifier, MLPClassifier, AdaBoostClassifier functions of the sklearn package (Pedregosa et al., 2011) in Python 3.1. To calculate acc, AUC scores and ROC-curves, we used the roc_curve, auc, and accuracy_score functions of the sklearn metrics module. We used the sklearn model_selection module to train and test classifier performance, search for the best parameters, and perform cross-validation.

### **Transfer learning techniques**

Since source and target datasets distributions differ (see Supplementary Fig. S2), direct predictions of deleterious mutations in target datasets using classifiers trained on source data could be biased. Therefore, we applied Transfer learning techniques to classify mutations in target datasets. In order to transfer knowledge from classification of human mutations to similar tasks for other mammals, we applied two different approaches: Transductive transfer learning and Inductive transfer learning.

***Transductive transfer learning***

Transductive transfer learning assumes that the source data is labeled but target data is not. So, we did not use labels for the target data until final validation of the predictions. We tested and tuned classifiers on human datasets and the parameters that provided the best classifier performance were further used in transfer learning tasks (see Supplementary Table S5 for details). Each sample from the source data
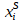
 was multiplied by weight $w_{i}$ that inversely depends on the distance from this sample to the mean of the cluster of the target data $a$:
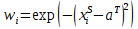


Therefore, samples from source data were taken into account only if they are close to the target data. This method prevents the negative transfer effect, i.e. the situation when samples from source data not connected with the target task are used thereby degrading the classifier performance. The classifiers are learned based on reweighted source data and tested on target data. To assess the performance of Transfer learning strategy, we compared its results with the results of direct classification without weights.

***Inductive transfer learning***

Inductive transfer learning assumes that source data and target data are labeled. Accordingly, we used labels for target data to construct a classifier and to test its performance. To provide independent test samples, we used cross-validation (see “The classifiers” subsection for a description of how the folds were made).

The Feature representation transfer is an instance of the Transductive transfer learning strategy that aims to transform the feature spaces of the source and target data to make them more similar and, thus, to decrease classification error. The new feature representation space for the target data was found by applying a neural network trained on the source data. The algorithm works as follows. First, we began with a neural network trained on source data, found parameters and weights, and fixed them. Next, the neural network was pruned at the last layer, which is just before the classification layer, and the target data was passed through the obtained network. Finally, the transformed target data in the new feature space were split into two parts, one for training and one for testing. Each classifier was trained on the training set and tested on the test set.

We used a three-layered network with 200, 200 and 15 nodes in the first, second and third layers, respectively. The rectifier activation function and hyperbolic tangent function were used for the first two layers and the last layer, respectively. The training and testing procedures were repeated 30 times in order to obtain statistically significant results. The Wilcoxon-Mann-Whitney test was applied to compare classification results of the target data in the initial feature space to that of the new space.

We validated the Inductive transfer learning approach on mouse data using the HumVar dataset as source data. The size of this dataset is sufficient to train a neural network. We used mouse data to validate this learning strategy as dog data are almost linearly separable and, therefore, can be predicted with a few samples. In such situations, information learned from a source domain may lead to a negative transfer effect.

***Implementation characteristics***

We used python 3.1 to write all the scripts and the sklearn package (Pedregosa et al., 2011) to implement machine learning tasks. Sample reweighting was performed with the standard argument “sample_weight” of the fit() attribute of the classifiers. A neural network was implemented using sknn (<https://github.com/aigamedev/scikit-neuralnetwork>). SIFT BLINK (Sim et al., 2012) was used as a standard in comparisons of the classifier accuracy developed on mouse and dog data. The implementation of Transfer learning algorithm is available as Supplementary code at [https://github.com/PlekhanovaElena/Transfer_learnin](https://github.com/PlekhanovaElena/Transfer_learning)g.

Note, that to classify different target datasets, one should retrain the Transductive classifier with weights computed from the distance between the source and the target data, which are different each time. However, training a classifier on human data with weights takes a few minutes: 5 minutes for HimDiv dataset and 15 minutes for HumVar dataset. In case of using Inductive classifier to classify different target dataset, we can train the neural network only once (it takes 2 hours for HumDiv and 8 hours for HumVar) and then pass each target dataset of interset through the obtained network (one pass takes about 15 minutes).

**Acknowledgments:** Our research has been supported by Zumberg and Waitt Foundations to SVN and by Russian science fund to SVN and MGS, grant 16-16-00007 (implementation of TL method and algorithm development). We also want to acknowledge Katrina Sherbina and Nathan Churches for comments on manuscript text.

## **Data archiving statement**

Data is available as Supplementary information (Supplementary table S1, S2, S3, S4). Implementation of Transfer learning algorithm and Supplementary tables S1 and S4 are available at [https://github.com/PlekhanovaElena/Transfer_learnin](https://github.com/PlekhanovaElena/Transfer_learning)g.

**Conflict of interest**

The authors declare no conflict of interests or competing financial interests.

## **References**

Adzhubei, I. A., Schmidt, S., Peshkin, L., Ramensky, V. E., Gerasimova, A., Bork, P., … Sunyaev, S. R. (2010). A method and server for predicting damaging missense mutations. *Nature Methods*, *7*(4), 248–9. https://doi.org/10.1038/nmeth0410-248

Altschul, S. F., Madden, T. L., Schäffer, A. A., Zhang, J., Zhang, Z., Miller, W., & Lipman, D. J. (1997). Gapped BLAST and PSI-BLAST: A new generation of protein database search programs. *Nucleic Acids Research*. https://doi.org/10.1093/nar/25.17.3389

Berman, H. M., Westbrook, J., Feng, Z., Gilliland, G., Bhat, T. N., Weissig, H., … Bourne, P. E. (2000). The Protein Data Bank. *Nucleic Acids Research*, *28*(1), 235–242. https://doi.org/10.1093/nar/28.1.235

Bi, J., Xiong, T., Yu, S., Dundar, M., & Rao, R. (2008). An Improved Multi-task Learning Approach with Applications in Medical Diagnosis. *Machine Learning and Knowledge Discovery in Databases*, 117–132. https://doi.org/10.1007/978-3-540-87479-9_26

Bovine HapMap Consortium, T. B. H., Gibbs, R. A., Taylor, J. F., Van Tassell, C. P., Barendse, W., Eversole, K. A., … Dodds, K. G. (2009). Genome-wide survey of SNP variation uncovers the genetic structure of cattle breeds. *Science (New York, N.Y.)*, *324*(5926), 528–32. https://doi.org/10.1126/science.1167936

Breiman, L. (2001). Random Forests. *Machine Learning*, *45*(1), 5–32. https://doi.org/10.1023/A:1010933404324

Capriotti, E., Calabrese, R., & Casadio, R. (2006). Predicting the insurgence of human genetic diseases associated to single point protein mutations with support vector machines and evolutionary information. *Bioinformatics*, *22*(22), 2729–2734. https://doi.org/10.1093/bioinformatics/btl423

Chasman, D., & Adams, R. M. (2001). Predicting the functional consequences of non-synonymous single nucleotide polymorphisms: structure-based assessment of amino acid variation11Edited by F. Cohen. *Journal of Molecular Biology*, *307*(2), 683–706. https://doi.org/10.1006/jmbi.2001.4510

Chattopadhyay, R., Sun, Q., Fan, W., Davidson, I., Panchanathan, S., & Ye, J. (2012). Multisource domain adaptation and its application to early detection of fatigue. *ACM Transactions on Knowledge Discovery from Data*, *6*(4), 1–26. <https://doi.org/10.1145/2382577.2382582>

Charlesworth, B. (2012). The effects of deleterious mutations on evolution at linked sites. *Genetics*, *190*(1), 5–22. https://doi.org/10.1534/genetics.111.134288

Chen, A. H., & Huang, Z.-W. (2010). A New Multi-Task Learning Technique to Predict Classification of Leukemia and Prostate Cancer (pp. 11–20). Springer, Berlin, Heidelberg. https://doi.org/10.1007/978-3-642-13923-9_2

Chun, S., & Fay, J. C. (2009). Identification of deleterious mutations within three human genomes. *Genome Research*, *19*(9), 1553–61. https://doi.org/10.1101/gr.092619.109

Consortium, U. (2017). UniProt: the universal protein knowledgebase. *Nucleic Acids Research*, *45*(D1), D158–D169. https://doi.org/10.1093/nar/gkw1099

Cortes, C., & Vapnik, V. (1995). Support-Vector Networks. *Machine Learning*, *20*(3), 273–297. https://doi.org/10.1023/A:1022627411411

Di, Y. M., Chan, E., Wei, M. Q., Liu, J.-P., & Zhou, S.-F. (2009). Prediction of deleterious non-synonymous single-nucleotide polymorphisms of human uridine diphosphate glucuronosyltransferase genes. *The AAPS Journal*, *11*(3), 469–80. https://doi.org/10.1208/s12248-009-9126-z

Eppig, J. T., Smith, C. L., Blake, J. A., Ringwald, M., Kadin, J. A., Richardson, J. E., & Bult, C. J. (2017). Mouse Genome Informatics (MGI): Resources for Mining Mouse Genetic, Genomic, and Biological Data in Support of Primary and Translational Research. In *Methods in molecular biology (Clifton, N.J.)* (Vol. 1488, pp. 47–73). https://doi.org/10.1007/978-1-4939-6427-7_3

Finn, R. D., Coggill, P., Eberhardt, R. Y., Eddy, S. R., Mistry, J., Mitchell, A. L., … Bateman, A. (2016). The Pfam protein families database: towards a more sustainable future. *Nucleic Acids Research*, *44*(D1), D279-85. https://doi.org/10.1093/nar/gkv1344

Freund, Y., & Schapire, R. E. (1999). A Short Introduction to Boosting. *Journal of Japanese Society for Artificial Intelligence*, *14*(5), 771–780.

Glorot, X., Bordes, A., & Bengio, Y. (2011). Domain Adaptation for Large-Scale Sentiment Classification: A Deep Learning Approach.

Grantham, R. (1974). Amino Acid Difference Formula to Help Explain Protein Evolution. *Science*, *185*(4154).

Hartfield, M., Glémin, S., Yu, J., & Purugganan, M. D. (2014). Hitchhiking of deleterious alleles and the cost of adaptation in partially selfing species. *Genetics*, *196*(1), 281–93. https://doi.org/10.1534/genetics.113.158196

Henikoff, S., & Henikoff, J. G. (1992). Amino acid substitution matrices from protein blocks. *Proceedings of the National Academy of Sciences of the United States of America*, *89*(22), 10915–9.

Huber, C. D., Kim, B. Y., Marsden, C. D., & Lohmueller, K. E. (2017). Determining the factors driving selective effects of new nonsynonymous mutations. *Proceedings of the National Academy of Sciences of the United States of America*, *114*(17), 4465–4470. https://doi.org/10.1073/pnas.1619508114

Jantzen, J. (1998). Introduction To Perceptron Networks. *Neural Networks*, *873*(98), 1–32.

Kaiser, V. B., & Charlesworth, B. (2009). The effects of deleterious mutations on evolution in non-recombining genomes. *Trends in Genetics : TIG*, *25*(1), 9–12. https://doi.org/10.1016/j.tig.2008.10.009

Kan, M., Wu, J., Shan, S., & Chen, X. (2014). Domain Adaptation for Face Recognition: Targetize Source Domain Bridged by Common Subspace. *International Journal of Computer Vision*, *109*(1–2), 94–109. https://doi.org/10.1007/s11263-013-0693-1

Khalaj, M., Abbasi, A. R., Shimojo, K., Moritomo, Y., Yoneda, K., & Kunieda, T. (2009). A missense mutation (p.Leu2153His) of the *factor VIII* gene causes cattle haemophilia A. *Animal Genetics*, *40*(5), 763–765. https://doi.org/10.1111/j.1365-2052.2009.01906.x

Kibbe, W. A., Arze, C., Felix, V., Mitraka, E., Bolton, E., Fu, G., … Schriml, L. M. (2015). Disease Ontology 2015 update: an expanded and updated database of human diseases for linking biomedical knowledge through disease data. *Nucleic Acids Research*, *43*(Database issue), D1071-8. <https://doi.org/10.1093/nar/gku1011>

Kim, B. Y., Huber, C. D., & Lohmueller, K. E. (2017). Inference of the Distribution of Selection Coefficients for New Nonsynonymous Mutations Using Large Samples. *Genetics*, *206*(1), 345–361. https://doi.org/10.1534/genetics.116.197145

Knol, E. F., Nielsen, B., & Knap, P. W. (2016). Genomic selection in commercial pig breeding. *Animal Frontiers*, *6*(1), 15. https://doi.org/10.2527/af.2016-0003

Kondrashov, A. (2012). Genetics: The rate of human mutation. *Nature*, *488*(7412), 467–468. https://doi.org/10.1038/488467a

Kowarsch, A., Fuchs, A., Frishman, D., & Pagel, P. (2010). Correlated mutations: a hallmark of phenotypic amino acid substitutions. *PLoS Computational Biology*, *6*(9). https://doi.org/10.1371/journal.pcbi.1000923

Lenffer, J., Nicholas, F. W., Castle, K., Rao, A., Gregory, S., Poidinger, M., … Ranganathan, S. (2006). OMIA (Online Mendelian Inheritance in Animals): an enhanced platform and integration into the Entrez search interface at NCBI. *Nucleic Acids Research*, *34*(Database issue), D599-601. https://doi.org/10.1093/nar/gkj152

Ma, Y., Gong, W., & Mao, F. (2015). Transfer learning used to analyze the dynamic evolution of the dust aerosol. *Journal of Quantitative Spectroscopy and Radiative Transfer*, *153*, 119–130. https://doi.org/10.1016/j.jqsrt.2014.09.025

MacArthur, J., Bowler, E., Cerezo, M., Gil, L., Hall, P., Hastings, E., … Parkinson, H. (2017). The new NHGRI-EBI Catalog of published genome-wide association studies (GWAS Catalog). *Nucleic Acids Research*, *45*(D1), D896–D901. <https://doi.org/10.1093/nar/gkw1133>

Ng, P. C., & Henikoff, S. (2001). Predicting Deleterious Amino Acid Substitutions. *Genome Research*, *11*(5), 863–874. https://doi.org/10.1101/gr.176601

Oquab, M., Bottou, L., Laptev, I., & Sivic, J. (2014). Learning and Transferring Mid-Level Image Representations using Convolutional Neural Networks. *IEEE Conference on Computer Vision and Recognition*, 911179–1.

Pabinger, S., Dander, A., Fischer, M., Snajder, R., Sperk, M., Efremova, M., … Trajanoski, Z. (2014). A survey of tools for variant analysis of next-generation genome sequencing data. *Briefings in Bioinformatics*, *15*(2), 256–78. https://doi.org/10.1093/bib/bbs086

Pausch, H., Schwarzenbacher, H., Burgstaller, J., Flisikowski, K., Wurmser, C., Jansen, S., … Fries, R. (2015). Homozygous haplotype deficiency reveals deleterious mutations compromising reproductive and rearing success in cattle. *BMC Genomics*, *16*(1), 312. https://doi.org/10.1186/s12864-015-1483-7

Pedregosa, F., Varoquaux, G., Gramfort, A., Michel, V., Thirion, B., Grisel, O., … Duchesnay, É. (2011). Scikit-learn: Machine Learning in Python. *Journal of Machine Learning Research*, *12*(Oct), 2825–2830.

Perlich, C., Dalessandro, B., Stitelman, O., Raeder, T., & Provost, F. (2013). Machine Learning for Targeted Display Advertising: Transfer Learning in Action.

Puniyani, K., Kim, S., & Xing, E. P. (2010). Multi-population GWA mapping via multi-task regularized regression. *Bioinformatics*, *26*(12), i208–i216. https://doi.org/10.1093/bioinformatics/btq191

Ramensky, V., Bork, P., & Sunyaev, S. (2002). Human non-synonymous SNPs: server and survey. *Nucleic Acids Research*, *30*(17), 3894–900.

Raszek, M. M., Guan, L. L., & Plastow, G. S. (2016). Use of Genomic Tools to Improve Cattle Health in the Context of Infectious Diseases. *Frontiers in Genetics*, *7*, 30. https://doi.org/10.3389/fgene.2016.00030

Schweikert, G., Schweikert, G., & Widmer, C. (2008). An empirical analysis of domain adaptation algorithms for genomic sequence analysis. *IN CONF. ON NEURAL INF. PROC. SYS. (NIPS*.

Sievers, F., Wilm, A., Dineen, D., Gibson, T. J., Karplus, K., Li, W., … Higgins, D. G. (2014). Fast, scalable generation of high-quality protein multiple sequence alignments using Clustal Omega. *Molecular Systems Biology*, *7*(1), 539–539. https://doi.org/10.1038/msb.2011.75

Sim, N.-L., Kumar, P., Hu, J., Henikoff, S., Schneider, G., & Ng, P. C. (2012). SIFT web server: predicting effects of amino acid substitutions on proteins. *Nucleic Acids Research*, *40*(W1), W452–W457. https://doi.org/10.1093/nar/gks539

Stachowicz, K., Sargolzaei, M., Miglior, F., & Schenkel, F. S. (2011). Rates of inbreeding and genetic diversity in Canadian Holstein and Jersey cattle. *Journal of Dairy Science*, *94*(10), 5160–5175. https://doi.org/10.3168/jds.2010-3308

Stoltzfus, A., Norris, R. W., DW, H., DA, P., K, Z., YP, Z., … RG, C. (2016). On the Causes of Evolutionary Transition:Transversion Bias. *Molecular Biology and Evolution*, *33*(3), 595–602. https://doi.org/10.1093/molbev/msv274

Stone, E. A., & Sidow, A. (2005). Physicochemical constraint violation by missense substitutions mediates impairment of protein function and disease severity. *Genome Research*, *15*(7), 978–986. https://doi.org/10.1101/gr.3804205

Sunyaev, S. R., Eisenhaber, F., Rodchenkov, I. V, Eisenhaber, B., Tumanyan, V. G., & Kuznetsov, E. N. (1999). PSIC: profile extraction from sequence alignments with position-specific counts of independent observations. *Protein Engineering*, *12*(5), 387–94.

Weiss, K., Khoshgoftaar, T. M., & Wang, D. (2016). A survey of transfer learning. *Journal of Big Data*, *3*(1), 9. https://doi.org/10.1186/s40537-016-0043-6

Widmer, C., Leiva, J., Altun, Y., & Rätsch, G. (2010). Leveraging Sequence Classification by Taxonomy-Based Multitask Learning (pp. 522–534). Springer, Berlin, Heidelberg. https://doi.org/10.1007/978-3-642-12683-3_34

Wilcoxon, F. (1945). Individual Comparisons by Ranking Methods. *Biometrics Bulletin*, *1*(6), 80–83.

Xu, Q., Xue, H., & Yang, Q. (2011). Multi-platform gene-expression mining and marker gene analysis. *International Journal of Data Mining and Bioinformatics*, *5*(5), 485–503.

Xu, Y., Pan, S. J., Xiong, H., Wu, Q., Luo, R., Min, H., & Song, H. (2017). A Unified Framework for Metric Transfer Learning. *IEEE Transactions on Knowledge and Data Engineering*, *29*(6), 1158–1171. https://doi.org/10.1109/TKDE.2017.2669193

Zhou, Z.-H., & Feng, J. (2017). Deep Forest: Towards An Alternative to Deep Neural Networks.

**Figure legends**

**Figure 1. ROC-curves for different classifiers, trained on HumDiv data.** Values of quality metrics ordered by decreasing AUC values are shown adjacent to the classifier name. The dotted line corresponds to the ROC-curve for random guessing. The inset zooms in on the left upper quadrant to better distinguish the ROC-curves.
