# Supplementary figures and images for: Prediction of deleterious mutations in coding regions of mammals with transfer learning
Source: Evol Appl. 2018 May 9;12(1):18–28. doi: 10.1111/eva.12607 (PMC6304693; doi:10.1111/eva.12607)

# Roc curves of HumDiv dataset

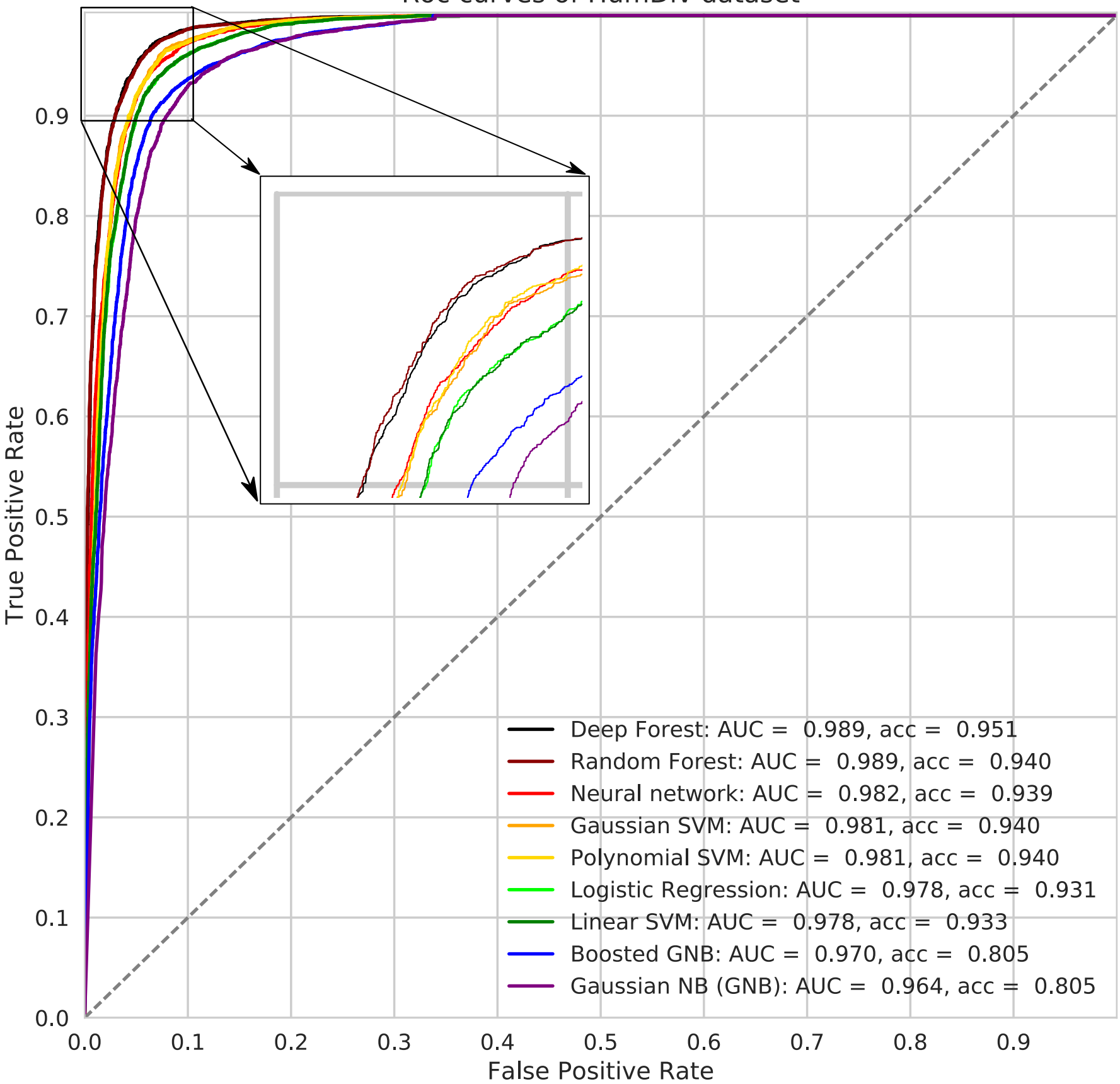

Supplement: Supplementary file 5 [file EVA-12-18-s005.zip › Transfer learning paper/Figure1.pdf]
