# Supplementary material for: Prediction of deleterious mutations in coding regions of mammals with transfer learning
Source: Evol Appl. 2018 May 9;12(1):18–28. doi: 10.1111/eva.12607 (PMC6304693; doi:10.1111/eva.12607)
Supplement: Supplementary file 5 [file EVA-12-18-s005.zip › Transfer learning paper/Cover_letter.doc]

**Cover letter**

Elena Plekhanova
plekhanova-elena@yandex.ru

15.10.2017

Dear Editor,

We wish to submit the manuscript entitled “Deleterious mutations in coding regions of mammals are more specifically recognized with transfer learning” for consideration by the Evolutionary Applications journal.

Our research is important in both scientific and application sides giving a method of classification mammalian deleterious from neutral amino acid substitutions. We use “transfer learning”, a set of machine learning techniques that provides a way to use known information about previous tasks in new ones, and, for the first time, demonstrate it’s potential to resolve this task. Because our findings are interesting from basic science and methodological points, we think they will attract broad readership. But, also they could be applied in the selection’s future improvement, that will be based on the genetic selection and efforts by thousands of farmers improving those over millennia. We believe our message will have followers, and ignite great interest in your journal audience.

More technically, in this paper, we employed the approaches of ‘transfer learning’ to take advantage of sizable human data sets by enabling their unbiased use in farm species by accounting for distances between human and mammals datasets. After validation of this approach using mouse and dog datasets with known mutation effects, we predict a number of deleterious cattle mutations breeding programs improvement can be based upon.

All raw data underlying the main results of the study is available as supplementary information.

We confirm that all authors approved the manuscript and its submission to the journal. We assert that all authors have seen and agreed to the submitted version of the manuscript.

We confirm that this work is original and has not been published elsewhere nor is it currently under consideration for publication elsewhere.

Please address all correspondence concerning this manuscript to me at plekhanova-elena@yandex.ru.

Thank you for receiving our manuscript and considering it for review. We appreciate your time and look forward to your response.

Sincerely,

Elena Plekhanova
